# Myogenesis modelled by human pluripotent stem cells: a multi‐omic study of Duchenne myopathy early onset

**DOI:** 10.1002/jcsm.12665

**Published:** 2021-02-14

**Authors:** Virginie Mournetas, Emmanuelle Massouridès, Jean‐Baptiste Dupont, Etienne Kornobis, Hélène Polvèche, Margot Jarrige, Alan R.L. Dorval, Maxime R.F. Gosselin, Antigoni Manousopoulou, Spiros D. Garbis, Dariusz C. Górecki, Christian Pinset

**Affiliations:** ^1^ INSERM UEVE UMR861, I‐STEM, AFM Corbeil‐Essonnes France; ^2^ CECS, I‐STEM, AFM Corbeil‐Essonnes France; ^3^ Biomics, C2RT Institut Pasteur Paris France; ^4^ Hub de Bioinformatique et Biostatistique ‐ Département BiologieComputationnelle Paris France; ^5^ Molecular Medicine, School of Pharmacy and Biomedical Sciences University of Portsmouth Portsmouth UK; ^6^ Department of Immuno‐Oncology Beckman Research Institute, City of Hope National Medical Center Duarte CA USA; ^7^ Unit for Cancer Sciences, Centre for Proteomics Research, Institute for Life Sciences University of Southampton Southampton UK; ^8^ Proteas Bioanalytics Inc. BioLabs at The Lundquist Institute Torrance CA USA; ^9^ Military Institute of Hygiene and Epidemiology Warsaw Poland; ^10^ CNRS, I‐STEM, AFM Corbeil‐Essonnes France

**Keywords:** Duchenne muscular dystrophy, Myogenesis, Human pluripotent stem cells, Omics

## Abstract

**Background:**

Duchenne muscular dystrophy (DMD) causes severe disability of children and death of young men, with an incidence of approximately 1/5000 male births. Symptoms appear in early childhood, with a diagnosis made mostly around 4 years old, a time where the amount of muscle damage is already significant, preventing early therapeutic interventions that could be more efficient at halting disease progression. In the meantime, the precise moment at which disease phenotypes arise—even asymptomatically—is still unknown. Thus, there is a critical need to better define DMD onset as well as its first manifestations, which could help identify early disease biomarkers and novel therapeutic targets.

**Methods:**

We have used both human tissue‐derived myoblasts and human induced pluripotent stem cells (hiPSCs) from DMD patients to model skeletal myogenesis and compared their differentiation dynamics with that of healthy control cells by a comprehensive multi‐omic analysis at seven time points. Results were strengthened with the analysis of isogenic CRISPR‐edited human embryonic stem cells and through comparisons against published transcriptomic and proteomic datasets from human DMD muscles. The study was completed with DMD knockdown/rescue experiments in hiPSC‐derived skeletal muscle progenitor cells and adenosine triphosphate measurement in hiPSC‐derived myotubes.

**Results:**

Transcriptome and miRnome comparisons combined with protein analyses demonstrated that hiPSC differentiation (i) leads to embryonic/foetal myotubes that mimic described DMD phenotypes at the differentiation endpoint and (ii) homogeneously and robustly recapitulates key developmental steps—mesoderm, somite, and skeletal muscle. Starting at the somite stage, DMD dysregulations concerned almost 10% of the transcriptome. These include mitochondrial genes whose dysregulations escalate during differentiation. We also describe fibrosis as an intrinsic feature of DMD skeletal muscle cells that begins early during myogenesis. All the omics data are available online for exploration through a graphical interface at https://muscle‐dmd.omics.ovh/.

**Conclusions:**

Our data argue for an early developmental manifestation of DMD whose onset is triggered before the entry into the skeletal muscle compartment, data leading to a necessary reconsideration of dystrophin roles during muscle development. This hiPSC model of skeletal muscle differentiation offers the possibility to explore these functions as well as find earlier DMD biomarkers and therapeutic targets.

## Introduction

Duchenne muscular dystrophy (DMD) is a rare genetic disease, but it is the most common form of myopathy affecting approximately one in 5000 male births and very rarely female. In this recessive X‐linked monogenic disorder, mutations in the *DMD* gene lead to the loss of functional dystrophin protein, resulting in a progressive—yet severe—muscle wasting phenotype.^1^ In patients, symptoms usually appear in early childhood (2–5 years old) and worsen with age, imposing the use of wheelchair before 15 and leading to premature death by cardiac and/or respiratory failure(s) mostly around 30 years of age.^2^

At the age of diagnosis (mostly around 4 years old), muscles of DMD patients have already suffered from the pathology.^3^, ^4^ Several reviews pointed out the limitations of current disease biomarkers, which fail to detect the development of DMD specifically and at an early age.^5^, ^6^ Meanwhile, no treatment is available to stop this degenerative disease yet. Developing therapies aim at restoring the expression of dystrophin in muscle cells, but so far, the level stays too low to be beneficial to patients.^7^ The absence of both reliable biomarkers and effective therapies stresses the need of better defining the first steps of DMD in humans to be able to increase diagnosis sensitivity and therefore improve patient management by accelerating their access to better healthcare as well as develop alternative therapeutic approaches by finding targets that compensate the lack of dystrophin and complement current attempts at restoring its expression.^8^

In 2007, a seminal publication reported that the gene expression profile of muscles from asymptomatic DMD children younger than 2 years old is already distinguishable from healthy muscles, suggesting that DMD molecular dysregulations appear before disease symptomatic manifestations.^4^ Evidence obtained in multiple animal models, such as neonatal *GRMD* dogs,^9^ DMD zebrafish,^10^ and *mdx* mouse embryos,^11^ as well as in human foetuses,^12^, ^13^, ^14^ even suggest that DMD starts before birth, during prenatal development. Our team recently identified the embryonic dystrophin isoform Dp412e expressed in early mesoderm‐committed cells,^15^ another indication that DMD can start *in utero*. Further exploring DMD onset in human foetuses is extremely challenging for obvious ethical and practical reasons. A way to overcome these issues is to develop a human DMD model *in vitro*, recapitulating embryonic development from human pluripotent stem cells to skeletal muscle lineage.

To our knowledge, none of the existing human DMD *in vitro* models, either based on tissue‐derived myoblasts^16^ or on the differentiation of induced pluripotent stem cells,^17^, ^18^, ^19^, ^20^, ^21^ have been used for studying DMD during the ontogeny of the skeletal muscle lineage. Moreover, original protocols for *in vitro* myogenesis from human pluripotent stem cells (reviewed in Kodaka *et al*.^22^) use transgene overexpression or/and cell sorting procedures and thereby miss the steps preceding skeletal muscle commitment, for example, paraxial mesoderm and myotome. Novel protocols have recently used transgene‐free directed differentiation to recapitulate human embryonic development in a dish, giving theoretical access to the developmental steps.^19^, ^23^, ^24^, ^25^

Using one of these protocols,^23^ we compared the myogenic differentiation dynamics of healthy and DMD human induced pluripotent stem cells (hiPSCs) using a multi‐omic approach to identify early disease manifestations *in vitro*. DMD cells showed marked transcriptome dysregulations from Day 10, before the detection of skeletal muscle regulatory factors at Day 17. Specifically, we identified the dysregulation of mitochondrial genes as one of the earliest detectable phenotypes. These alterations escalated over the course of muscle specification. In addition, we showed an early induction of Sonic hedgehog (SSH) signalling pathway, followed by collagens as well as fibrosis‐related genes suggesting the existence of an intrinsic fibrotic process solely driven by DMD muscle cells. Overall, our data highlight that human pluripotent stem cells are a suitable cell model to study the ontogeny of skeletal muscle lineage in both healthy and disease conditions. In the context of DMD, they strongly argue for the existence of early disease manifestations during somite development.

## Materials and methods

### Ethics, consent, and permissions

The collection of primary myoblasts was established from patient muscle biopsies at the Cochin Hospital/Cochin Institute as part of the medical diagnostic procedure of neuromuscular disorders. A signed and informed consent was obtained by the Cochin Hospital cell bank/Assistance Publique‐Hôpitaux de Paris for each patient included in this study to collect, establish, and study primary cultures of fibroblasts and myoblasts. This collection of myoblasts was declared to (i) legal and ethical authorities at the Ministry of Research (declaration number 701, modified declaration number 701–1) via the medical hosting institution (Assistance Publique‐Hôpitaux de Paris) and to (ii) the ‘Commission Nationale de l'Informatique et des Libertés’ (declaration number 1154515).

### Cells

Human primary adult myoblasts from healthy individuals and DMD patients were provided by Celogos laboratory and Cochin Hospital/Cochin Institute (Supporting Information, *Table* S1). DMD cells carry the following mutations: in‐frame duplication exons 3–26 (DMD Myoblast 1), out‐of‐frame deletion exons 8–43 (DMD Myoblast 2), and stop exon 7 c (DMD Myoblast 3). In Celogos laboratory, cell preparation was done according to patent US2010/018873 A1.

### Cell culture

#### Human tissue‐derived myoblasts

Primary myoblasts were maintained in a myoblast medium: DMEM/F‐12, HEPES (31330–038, Thermo Fisher Scientific) supplemented with 10% foetal bovine serum (Hyclone, Logan, UT), 10 ng/mL fibroblast growth factor 2 (FGF2, 100‐18B, Peprotech), and 50 nM dexamethasone (D4902, Sigma‐Aldrich) on 0.1% gelatin‐coated (G1393, Sigma‐Aldrich) cultureware.

#### Human tissue‐derived myotubes

Primary myoblasts were differentiated into myotubes. Cells were seeded at 600 cells/cm^2^ on 0.1% gelatin‐coated cultureware in myoblast medium containing 1 mM acid ascorbic 2P (A8960, Sigma‐Aldrich).

#### Human induced pluripotent and embryonic stem cells

Primary myoblasts were reprogrammed into hiPSCs following the protocol described in Massouridès *et al*.,^15^ using the Yamanaka's factors POU5F1, SOX2, cMYC and KLF4 transduction by ecotropic or amphotropic vectors (*Table* S1). HiPSCs and human embryonic stem cells (hESCs) were adapted and maintained with mTeSR™1 culture medium (05850, Stemcell Technologies) on Corning® Matrigel® Basement Membrane Matrix coated cultureware (354234, Corning Incorporated). Cells were then seeded at 20 000 cells/cm^2^, passaged, and thawed each time with 10 μM StemMACS™ Y27632.

#### Human embryonic stem cell‐derived and human induced pluripotent stem cell‐derived cells

*S*ix hiPSCs (three healthy and three DMD) were differentiated three times towards skeletal muscle lineage using commercial media designed from the work of Caron *et al*.^23^ (Skeletal Muscle Induction medium SKM01, Myoblast Cell Culture Medium SKM02, Myotube Cell Culture Medium SKM03, AMSbio). This protocol is a 2D directed differentiation that uses three consecutive defined media (SKM01 from Day 0 to 10, SKM02 from Day 10 to 17, and SKM03 from Day 17 to 25) and only one cell passage at Day 10. Cells were seeded at 3500 cells/cm^2^ at Day 0 and Day 10 on BioCoat™ Collagen I cultureware (356485, Corning Incorporated). Part of the cell culture was frozen at Day 17 for further experiments such as DNA extraction. These cells were then thawed at 30 000 cells/cm^2^ and cultured in SKM02 for 3 days and SKM03 for 3 additional days to get myotubes. Two isogenic hESCs (one healthy and one DMD) were differentiated two times using the same protocol.

#### Gene editing

Exon 52 of the *DMD* gene was removed by gene editing. Benchling and Crispor software were used to design the single guide (sg) RNAs upstream and downstream of *DMD* exon 52 (sgRNA sequences are followed by scaffold sequences, which are underlined; upstream: GTTTGGTGATTCTTACGGACGTTTCAGAGCTATGCTGGAAACAGCATAGCAAGTTGAAATAAGGCTAGTCCGTTATCAACTTGAAAAAGTGGCACCGAGTCGGTGCTTTTTTT; downstream: GCCCACCCTACTACGGCATAGTTTCAGAGCTATGCTGGAAACAGCATAGCAAGTTGAAATAAGGCTAGTCCGTTATCAACTTGAAAAAGTGGCACCGAGTCGGTGCTTTTTTT). SgRNAs were diluted at 150 pmol/μL in TE 1X buffer and then at 30 pmol/μL in nuclease‐free water. The ribonucleoprotein complex was formed in 25 μL, with 20 pmol of Cas9 2NLS nuclease (S. pyrogenes, Synthego) mixed to 90 pmol of each diluted sgRNAs in P3 solution (V4XP‐3024, Lonza), incubated at room temperature for 10 min before being stored at 4°C. After an amplification in mTeSR™1 culture medium (05850, Stemcell Technologies) on Corning® Matrigel® Basement Membrane Matrix coated cultureware, SA001 hESCs (Cellartis) were harvested and passed through a sieve (40 μm, Falcon). A total of 150 000 cells were centrifuged for 5 min at 90 *g*, resuspended in 5 μL of P3 solution, and mixed to the complex. The resulting 30 μL were then transferred to a 16‐well Nucleocuvette™ Strip processed with the 4D‐Nucleofector System™ (Lonza) to introduce the complex into cells by electroporation (CD118 program). Cells were then seeded at a non‐clonal density in pre‐warmed mTeSR™1 culture medium with 10 μM Rock Inhibitor on Corning® Matrigel® Basement Membrane Matrix coated 24‐well cultureware. One well was used 48 h later to do DNA extraction (QuickExtract™ DNA, Lucigen) and validate the deletion by PCR [using Q5® High‐Fidelity DNA Polymerase (New England Biolabs) and DMD Delta exon 52 primers (*Table* S2); with the program: 95°C for 5 min; for 35 cycles: 95°C for 30 s, 64°C for 30 s, 72°C for 39 s; and 72°C for 2 min; amplicon of 893 pb]. Seventy‐six hours after the CRISPR, one well was used for cell banking and another was used to be seeded into 96‐well plates at a clonal density with cloneR (#05888, Stemcell Technologies) addition and a medium change after 2 days. The first half of each colony was used after 15 days of culture for amplification, while the other half was processed for PCR analysis. Eurofins Genomics performed the sequencing of the selected clone. The resulting sequence was analysed with Benchling, NCBI (BLASTn), and Biomanda: the DMD Delta52 cell line had a deletion of 1161 base pair (bp) containing the targeted exon 52 (118 pb) and an insertion of a random sequence of 6 bp.

### DNA and RNA experiments

#### RNA extraction and quality

RNA extraction was done in the six cell lines at seven different time points: tissue‐derived myoblast and tissue‐derived myotube, as well as during hiPSC differentiation at Days 0, 3, 10, 17, and 25 (hiPSC‐derived myotube) using the miRNeasy Mini kit (217004, QIAgen) on the QIAcube instrument. RNAs coming from part A of the extraction protocol was used for mRNA‐seq and RT‐qPCR. RNAs coming from part B of the extraction protocol was used for miRseq. PartA RNA was quantified by Nanodrop spectrophotometer (ND‐1000, Thermo Fisher Scientific) and purity/quality (RIN ≥ 7) was assessed with the 2200 TapeStation using the Agilent RNA ScreenTape (5067‐5576/5067‐5577/5067‐5578, Agilent). PartB RNA was quantified and purity/quality was assessed with the 2100 Agilent Bioanalyzer using the Agilent small RNA kit (5067‐1548, Agilent).

#### Reverse transcription

Total RNA (500 ng) were reverse transcribed with random primers (48190–011, Thermo Fisher Scientific), oligo (dT) (SO131, Thermo Fisher Scientific), and deoxynucleotide (dNTP, 10297–018, Thermo Fisher Scientific) using Superscript® III reverse transcriptase (18080–044, Thermo Fisher Scientific). Thermocycling conditions were 10 min, 25°C; 60 min, 55°C; and 15 min, 75°C.

#### Quantitative PCR

Total DNA and cDNA were amplified using primers (Thermo Fisher Scientific) designed with Primer blast tool (http://www.ncbi.nlm.nih.gov/tools/primer‐blast) and listed in *Table* S2. The amplification efficiency of each primer set was preliminarily determined by running a standard curve. Detection was performed using a QuantStudio™ 12K Flex Real‐Time PCR System (Thermo Fisher Scientific). Reactions were carried out in a 384‐well plate, with 10 μL containing 2.5 μL of 1/10 cDNA or 6.25 ng/uL total DNA, 0.2 μL of mixed forward and reverse primers at 10 μM each, and 5 μL of 2X Luminaris Color HiGreen qPCR Master Mix Low Rox (K0973, Thermo Fisher Scientific). Thermocycling conditions were 50°C during 2 min and 95°C during 10 min, followed by 45 cycles including 15 s at 95°C and 1 min at 60°C plus a dissociation stage. All samples were measured in triplicate. Experiments were normalized using UBC as reference gene and relative quantification was done with the ΔΔCt method.

#### Bulk mRNA sequencing

Libraries were prepared with the TruSeq Stranded mRNA kit protocol according to supplier recommendations. Briefly, the first step was the purification of PolyA‐containing mRNA molecules from 1 μg of total RNA using poly‐T oligomers attached to magnetic beads, followed by a fragmentation using divalent cations under elevated temperature to obtain approximately 300 bp fragments. Then, double‐stranded cDNA was synthesized, Illumina adapters were ligated, and the cDNA library was amplified by PCR for sequencing. Finally, paired‐end 100 bp/75 bp sequencing was carried out with an Illumina HiSeq 4000 sequencer.

The RNA‐seq analysis workflow was designed using snakemake 3.5.4^26^ for read quality estimation, mapping, and differential expression analysis. Quality estimation was obtained with FastQC 0.11.5 (https://www.bioinformatics.babraham.ac.uk/projects/fastqc/). Mapping to the human genome assembly Ensembl GRCh37.87 (43 695 transcripts) was performed with STAR 2.5.0a.^27^ According to STAR manual and for more sensitive novel junction discovery, the junctions detected in a first round of mapping were used in a second mapping round. Read strandness was confirmed using RSeQC.^28^ Analysis results were summarized using MultiQC 1.0.^29^ Normalized counts (median ratio normalization) and differential expression analysis were performed with DESeq2 1.16.1,^30^ considering pairwise comparisons with all developmental stages and comparing DMD vs. healthy cells within developmental stages. BiomaRt 2.30.0^31^ was used to fetch gene annotations from Ensembl. Transcripts with |log2FoldChange| ≥ 0.4 (equivalent of DMD/healthy ratio ≤0.76 or ≥1.32) and adjusted *P* value ≤ 0.05 were considered differentially expressed. RNA‐seq data have been deposited in the ArrayExpress database^32^ at EMBL‐EBI under accession number E‐MTAB‐8321 (https://www.ebi.ac.uk/arrayexpress/experiments/E‐MTAB‐8321).

#### Single‐cell mRNA sequencing

One cryotube (1.7 million cells) of the M398 healthy cell line was thawed in a T75 BioCoat™ Collagen I cultureware (356485, Corning Incorporated) with SKM02 medium (AMSbio). Cells were harvested with trypsin the following day. A total of 1.5 million cells were centrifuged at 300 *g* for 5 min. Cells were resuspended at 1500 cells/μL in DMEM F‐12 with 10% serum and processed according to the 10X Genomics single‐cell RNA sequencing protocol. Briefly, the library was prepared with Chromium™ Single Cell 3' Library & Gel Bead Kit v2. Six thousand cells with a viability of 94% were processed to finally sequence 3625 of them. Sequencing was done on Illumina HiSeq 4000 sequencer. Bioinformatics analysis was performed using 10X Genomics recommendations. Briefly, fastq files were created with cellranger mkfastq, and cellranger count was used to perform alignment, filtering, barcode counting, and unique molecular identifier counting. RNA‐seq data have been deposited in the ArrayExpress database^32^ at EMBL‐EBI under accession number E‐MTAB‐9510 (https://www.ebi.ac.uk/arrayexpress/experiments/MTAB‐9510).

#### miRNA sequencing

miRNAs (10 ng) were reverse transcribed using the Ion Total RNA‐seq kit v2 (4475936, Thermo Fisher Scientific) following the manufacturer's protocol for small RNA libraries. The cDNA libraries were amplified and barcoded using Ion Total RNA‐seq kit v2 and Ion Xpress RNA‐seq Barcode Adapters 1‐16 Kit (Thermo Fisher Scientific). Amplicons were quantified using Agilent High Sensitivity DNA kit before samples pooling in sets of 15. Emulsion PCR and enrichment were performed on the Ion OT2 system instrument using the Ion PI Hi‐Q OT2 200 kit (A26434, Thermo Fisher Scientific). Samples were loaded on an Ion PI v3 Chip and sequenced on the Ion Proton System using Ion PI Hi‐Q sequencing 200 kit chemistry (200 bp read length; A26433, Thermo Fisher Scientific). Sequencing reads were trimmed with Prinseq^33^ (v0.20.4) (‐‐trim‐right 20) and filtered by average quality score (‐‐trim‐qual 20). Reads with a size less than 15 bp were removed and reads with a size greater than 100 bp were trimmed with Cutadapt (v1.16).^34^ Mapping to the human genome assembly Ensembl GRCh37.87 (3111 transcripts) was performed with STAR 2.5.3a.^27^ Normalized counts (median ratio normalization) and differential expression analysis were performed with DESeq2 1.16.1,^30^ considering pairwise comparisons with all developmental stages and comparing DMD vs. healthy cells within developmental stages. Transcripts with |log2FoldChange| ≥ 0.4 (equivalent of DMD/healthy ratio ≤0.76 or ≥1.32) and *P* value ≤ 0.05 were considered differentially expressed. The use of *P* value instead of adjusted *P* value was justified by biological meaning^35^ (i.e. well‐known regulated/dysregulated miRNAs had a *P* value ≤ 0.05 but not an adjusted *P* value ≤ 0.05). miRNA‐seq data have been deposited in the ArrayExpress database^32^ at EMBL‐EBI under accession number E‐MTAB‐8293 (https://www.ebi.ac.uk/arrayexpress/experiments/E‐MTAB‐8293).

#### High‐throughput data analyses

Graphs were realized using RStudio. Viridis 0.5.1 library^36^ was used for the colour palette to ease reading with colour blindness and print well in grey scale. Normalized counts were standardized for unsupervised analyses with the scale function (centre = TRUE, scale = TRUE) and plotted with the corrplot function from corrplot 0.84 library.^37^ Spearman correlation was done with the cor function (method = ‘spearman’, use = ‘pairwise.complete.obs’) on standardized data. Hierarchical clustering and heatmap were performed with gplots 3.0.3 library^38^ heatmap.2 function on standardized data. Gene enrichment data were retrieved from DAVID database using RDAVIDWebService 1.24.0 library^39^ on supervised list of mRNAs [mRNA‐seq data: adjusted *P* value ≤ 0.01, normalized counts ≥ 5 in at least one sample, ratio ≤ 0.5 or ≥2 for myogenesis (*Figure* S1B) and ratio ≤ 0.76 or ≥1.32 for DMD phenotype (*Figure* S1C); enrichment data: Benjamini value ≤ 0.05, enrichment ≥ 1.5]. Only Gene Ontology terms were processed. Spearman correlations for the transcriptomics vs. proteomics comparison at Day 17 and for comparisons with published omics datasets were performed using two‐tailed non‐parametric Spearman correlation by GraphPad Prism software.

#### Exon skipping

One million cells were transfected after 17 days of culture by electroporation with a phosphorodiamidate morpholino oligomer (PMO) targeting exon 7 (custom oligo PMO7 5′‐ATGTTGAATGCATGTTCCAGTCGTTGTGTG‐3′, Gene Tools) or 51 (custom oligo PMO51 5′‐CTCCAACATCAAGGAAGATGGCATTTCTAG‐3′, Gene Tools) of the *DMD* gene in 100 μL solution from the P3 Primary Cell 4D‐Nucleofector® X Kit L (V4XP‐3024, Lonza) using the CB150 program on the 4D‐Nucleofector™ System (Lonza). PMO7 was transfected into healthy M180 cells at concentrations of 0.1, 0.25, 0.5, 0.75, 1, 5, 10, or 100 μM. PMO51 was transfected into DMD52 cells at concentrations of 0.1, 0.5, 20, 50, 60, 75, or 100 μM. A PMO control (standard control 5′‐CCTCTTACCTCAGTTACAATTTATA‐3′, Gene Tools) was transfected at a concentration of 100 μM. Cells were seeded at a density of 100 000 cells/cm^2^. RNA extraction was carried on transfected cells 24, 48, and 72 h later followed by a reverse transcription as described above. PCR was done on 1 μL of cDNA using 10 μM of forward and reverse primers (*Table* S2, Life technologies) and 1 unit of Taq DNA polymerase (10342, Life technologies) as described by the manufacturer's instructions, for a final reaction volume of 25 μL. PCR reaction started by a step at 94°C for 3 min, followed by 27 cycles at 94°C for 45 s, 55°C for 45 s, and 72°C for 45 s and a final step at 72°C for 5 min. Exon skipping was analysed using the DNA 1000 kit (5067, Agilent) with the Agilent 2100 Bioanalyzer. Full‐length PCR product was 372 bp and exon skipped length PCR product was 253 bp for M180 cells and 422 bp and 189 bp for DMD52 cells. Results were computed by the Agilent 2100 Bioanalyzer software v3.81. Spearman correlations were performed using two‐tailed non‐parametric Spearman correlation by GraphPad Prism software.

### Protein experiments

#### Immunolabelling

Cells (healthy hiPSC 1/DMD hiPSC 2, *Table* S1) at Day 17 of culture were thawed and seeded at 10 000 cells/cm^2^ in SKM02 medium in Falcon® 96‐well microplate (353219, Corning) coated with 0.1% gelatin (G1393, Sigma‐Aldrich) and 2.4 μg/mL laminin (23017015, Thermo Fisher Scientific) in phosphate‐buffered saline (PBS) 1X (D8537, Sigma‐Aldrich). Cells were switched to DMEM/F‐12, HEPES (31330038, Thermo Fisher Scientific) with 2% horse serum (H1270, Sigma‐Aldrich) after 4 days of culture. Cells were fixed 15 min at 4°C with paraformaldehyde 4% (15710, Euromedex) after 7 days of culture. A first quick PBS 1X tablets (P4417, Sigma‐Aldrich) wash was done, followed by another lasting 10 min. Then, a solution with PBS 1X, Triton™ X‐100 0.25% (T8787, Sigma‐Aldrich) and bovine serum albumin 2.5% (A9418, Sigma‐Aldrich) was added and incubated 30 min at room temperature. Primary antibody diluted in the same buffer (α‐actinin 1/500, A7811, Sigma‐Aldrich) was finally added overnight at 4°C. Two quick PBS 1X washes followed by a third incubated 10 min at room temperature were done the next day. An incubation was done 45 min at room temperature with a mix of 4′,6‐diamidine‐2′‐phenylindole dihydrochloride (DAPI, 1 μg/mL, 10236276001, Sigma‐Aldrich) and the secondary antibody Donkey anti‐Mouse Alexa Fluor 555 in PBS 1X, (1/1000, A‐31570, Thermo Fisher Scientific). Finally, two quick PBS 1X washes were followed by a third incubated 10 min at room temperature. Stained cells were kept in PBS 1X at 4°C before imaging with a Zeiss LSM880 Airyscan confocal and Zen software (Black edition).

#### Western blotting

Culture of tissue‐derived myotubes were washed three times with cold PBS 1X (w/o Ca^2+^ and Mg^2+^, D8537, Sigma‐Aldrich) and proteins were isolated by scraping (010154, Dutscher) cultured cells with an extraction protein buffer [NaCl 150 mM, Tris 50 mM, EDTA 10 mM (AM9260G, Thermo Fisher Scientific), Triton 1X, 1/100 Protease Inhibitor Cocktail (P8340, Sigma‐Aldrich), PhosSTOP tablet (04906845001, Roche Diagnostics)]. Cell pellets of hiPSC‐derived myotubes were rinsed once with cold PBS 1X, spun 5 min at 300 *g*, and resuspended in the same extraction protein buffer. Protein extracts were centrifuged at 4°C 10 min at 16 000 *g* and supernatants were kept at −80°C. Quantitation of total protein was done with Pierce BCA protein assay kit (23225, Thermo Fisher Scientific). Protein extracts were mixed before gel loading with 9 μL of loading buffer [urea 4 M, sodium dodecyl sulfate (SDS) 3.8%, glycerol 20%, Tris 75 mM pH 6.8, 5% β‐mercaptoethanol, 0.1 mg/mL bromophenol blue] and completed with 28 μL of extraction protein buffer (for one well) and then heated once 5 min at 95°C. Western blots for RYR1 (1/1000, MA3‐925, Thermo Fisher Scientific), MF20 (1/500, DSHB, concentrate), Manex50 (1/30, DSHB), α‐sarcoglycane (1/150, A‐SARC‐L‐CE, Leica biosystems), and γ‐sarcoglycane (1/150, G‐SARC‐CE, Leica biosystems) were performed with Criterion™ XT Tris‐Acetate Precast Gels 3–8% (3450130, Bio‐Rad, Hercules, CA), XT Tricine running buffer (161–0790, Bio‐Rad) and ran at room temperature for 1 h and 15 min at 150 V. Western blots for CaV1.1 (1/1000, MA3‐920, Thermo Fisher Scientific), ATP5A (1/1,000, ab14748, ABCAM), Semaphorin 6A (1/55, AF1146, R&D systems), and GLI3 (1/200, AF3690, R&D systems) were performed with 4–15% Criterion™ TGX™ Precast Midi Protein Gel (5671084, Bio‐Rad), 10x Tris/Glycine/SDS Running Buffer (1610772), and ran at room temperature for 1 h at 200 V. Gels were rinsed once in water and blotted either with ‘high molecular weight’ or ‘mixed molecular weight’ program of Trans‐Blot® Turbo™ transfer system (Bio‐Rad) using Trans‐Blot® Turbo™ Midi Nitrocellulose Transfer Packs (170–4159, Bio‐Rad). Blots were then processed with the SNAP i.d.® 2.0 Protein Detection System following the manufacturer's protocol, using Odyssey® Blocking Buffer (927‐40003, LI‐COR) for blocking and 0,2% Tween® for antibody dilutions (28829.296, VWR). Washes were done with PBS tween buffer (PBS 1X tablets, P4417, Sigma‐Aldrich; 0.1% Tween® 20). Each primary antibody was pooled with either α‐actinin (1/12,500, sc‐17829, Santa Cruz or 1/7000, A7811, Sigma‐Aldrich) or α‐tubulin (1/6666, Ab7291, Abcam). Either IRDye 800CW donkey anti‐mouse and/or IRDye® 680RD donkey anti‐goat (1/5000‐1/10000, 926‐32212, 926‐68074, LI‐COR) were used as secondary antibodies. After completion of SNAP i.d.® general protocol, two PBS 1X washes were finally done with the membrane still in the blot holder before band visualization with Odyssey® CLx Imaging System and quantification with Image Studio Lite software (Version 5.2). Statistical analysis was performed using unpaired *t*‐test by GraphPad Prism software.

#### Tandem mass tag isobaric quantitative proteomics

##### Samples preparation

Cells were collected after 17 days of culture and resuspended in 90% foetal bovine serum (Hyclone), 10% DMSO (A3672.0050, VWR), cooled down until −90°C with the CryoMed™ device (Thermo Fisher Scientific), before storage in liquid nitrogen. Cells were then thawed and washed five times with cold PBS and air was replaced by Argon to thoroughly dry the pellet that was flash frozen in liquid nitrogen. Five to ten times the approximate cell pellet volume of 0.5 M triethyl ammonium bicarbonate with 0.05% SDS was added to the cell pellet for protein extraction. Cell pellet was resuspended and triturated by passing through a 23‐gauge needle and 1 mL syringe for 30 times. Samples were then sonicated on ice at amplitude of 20% for 30 × 2 s bursts and centrifuged at 16 000 *g* for 10 min at 4°C. Supernatant was transferred to a fresh Eppendorf tube. Protein was quantified by Nanodrop spectrophotometer. A total of 100–150 μg of protein was aliquoted for each individual sample and 2 μL TCEP (50 mM tris‐2‐carboxymethyl phosphine) was added for every 20 μL of protein used for reducing the samples. After 1 h of incubation at 60°C, 1 μL MMTS (200 mM methylmethane thiosulphonate) was added for every 20 μL of protein used for alkylating/‘blocking’ the samples. Finally, after a 10 min incubation at room temperature, samples were trypsinized by addition of 6–7.5 μL of 500 ng/μL trypsin. The ratio between enzyme:substrate was 1:40. Samples were incubated overnight at 37°C in the dark.

##### Tandem mass tag labelling

When tandem mass tag (TMT) reagents reached room temperature, 50 μL of isopropanol/[acetonitrile] was added to each TMT 11‐plex reagent and was incubated at room temperature for 2 h, in the dark; 8 μL of 5% hydroxylamine was added to neutralize the reaction. Each sample was separately lyophilized at 45°C. Samples have been stored at −20°C or used immediately.

##### Offline C4 high‐performance liquid chromatography

All eight samples were pooled together in 60 μL of 97% mobile phase A (99.92% % H_2_O, 0.08% NH_4_OH) and 3% mobile phase B (99.92% % acetonitrile, 0.02% NH_4_OH) by serially reconstituting each sample. Extra 40 μL of mobile phase was added to Sample 1; after sample has been well vortexed, its content was transferred to the tube with Sample 2 (and serially repeated until all samples were pooled). Final volume of samples needed to be 100 μL. After sample was centrifuged at 13 000 *g* for 10 min, supernatant was collected with a high‐performance liquid chromatography (HPLC) injection syringe; 100 μL was injected onto the sample loop. Fractions were collected in a peak‐dependent manner. Finally, fractions were lyophilized at 45°C and stored at −20°C until required. The used column was a Kromasil C4 column 100 Å pore size, 3.5 μm particle size, 2.1 mm inner diameter, and 150 mm length. The gradient for C4 separation was (RT in min—%B): 0‐3, 10‐3, 11‐5, 16‐5, 65‐20, 100‐30, 15‐80, 120‐80, and 125‐3.

##### Solid phase extraction cleaning of peptides fractions

A GracePureTMT SPE C18‐Aq cartridge was used for pre‐cleaning of samples (support: silica, % carbon: 12.5%, with endcapping, surface area: 518 m^2^/g, particle size: 50 μm, pore size: 60 Å, water‐wettable). Samples were reconstituted using in total 400 μL of 1% Formic Acid (ACN) and 0.01% formic acid (FA). Cartridge was washed with 600 μL of ACN. ACN was then completely flushed out of the column at dropwise speed. This activated the ligands. Then, 1% ACN and 0.01% FA (600 μL) was flushed through the cartridge to equilibrate the sorbent; 400 μL of the sample was loaded in the cartridge. It was then very slowly flushed through the cartridge and recovered into a fresh tube. This process was repeated three times. Two volumes of 250 μL of 1% ACN and 0.01% FA were used to clean and de‐salt the sample. It was flushed through very slowly. Two volumes (250 μL each) were used per step (2% ACN, 10% ACN, 30% ACN, 50% ACN, 70% ACN). This cycle was repeated twice. Each particular concentration was pooled in one tube. Samples were dried to dryness in a Speedvac at room temperature overnight and stored at −20°C. Like previously, samples were pooled with 100 μL of 97% mobile phase A (99.92% % H_2_O, 0.08% NH_4_OH) and 3% mobile phase B (99.92% acetonitrile, 0.02% NH_4_OH) and injected onto the sample loop. Fractions were collected in a peak‐dependent manner. The gradient for SPE‐cleaned peptides C4 separation (RT in min—%B): 0‐2, 10‐2, 20‐5, 25‐5, 35‐20, 55‐35, 60‐35, 70‐80, 75‐80, and 80‐3.

##### Online C18 high‐precision liquid chromatography

Thirty microliters of loading phase (2% acetonitrile, 1.0% formic acid) was added to each fraction‐containing Eppendorf tube. Samples were vortexed and centrifuged. Blanks (30 μL mobile phase) were added into wells A1 to A12. Thirty microliters of Sample 1 was pipetted into well B1, Sample 2 in well B2, and so on. An orthogonal 2D‐LC–MS/MS analysis was performed with the Dionex Ultimate 3000 UHPLC system coupled with the ultra‐high‐resolution nano ESI LTQ‐Velos Pro Orbitrap Elite mass spectrometer (Thermo Fisher Scientific).

##### Data analysis

Higher‐energy collisional dissociation (HCD) and collision‐induced dissociation (CID) tandem mass spectra were collected and submitted to Sequest search engine implemented on the Proteome Discoverer software version 1.4 for peptide and protein identifications. All spectra were searched against the UniProtKB SwissProt. The level of confidence for peptide identifications was estimated using the Percolator node with decoy database searching. False discovery rate was set to 0.05, and validation was based on the *q* value. Protein ratios were normalized to protein median and peptides with missing TMT values were rejected from protein quantification. Phosphorylation localization probability was estimated with the phosphoRS node. Protein ratios were transformed to log_2_ ratios and significant changes were determined by two‐tailed one‐sample *t*‐test with the Benjamini method for multiple testing corrections. To reduce the impact of possible false positive identifications, more parameters were set: (i) only proteins with more than two quantified unique peptides and (ii) DMD/healthy ratio ≥1.32 or ≤0.76 and 3 only false discovery rate corrected *P* value ≤ 0.05 were retained for bioinformatics analysis. The list of proteins quantified in the six samples is in *Table* S3. Proteomic data have been deposited in the PRIDE Archive database^40^ at EMBL‐EBI under accession number PXD015355 (https://www.ebi.ac.uk/pride/archive/projects/PXD015355).

### Adenosine triphosphate experiments

Two healthy (M180 and M398) and two DMD (M202 and M418) cell lines after 17 days of culture were seeded in 384‐well plates at a density of 30 000 cells/cm^2^. Living cells were stained with HOECHST at a concentration of 1/300 6 days later for cell quantification (nuclei per well were counted using the CX7 imaging system, Thermo Fisher Scientific). Adenosine triphosphate (ATP) measurement was done using the CellTiter‐Glo™ Luminescent Cell Viability Assay Kit (Promega) following the manufacturer's protocol and normalized by the cell quantification. Statistical analysis was performed using two‐tailed one‐sample *t*‐test by GraphPad Prism software (each healthy cell line was compared with each DMD cell line).

## Results

To establish the early/developmental impact of *DMD* gene mutations, hiPSCs from three DMD patients and three healthy individuals were generated as described previously.^15^ These cells were subjected to a standardized differentiation protocol without utilization of feeder cells, cell sorting, or gene overexpression resulting in elongated and plurinucleated myotubes within 25 days,^23^ with an amplification fold of 2918 ± 480 (mean ± SEM). Skeletal muscle progenitor cells after 10 and 17 days of differentiation could be cryopreserved (*Figure* S2A). Whole transcriptome and miRnome profiles were compared at seven differentiation time points (tissue‐derived myoblasts and myotubes, as well as hiPSC‐derived cells at Days 0, 3, 10, 17, and 25) and complemented by single‐cell transctriptomics, TMT proteomics and western blot analyses (*Table* S4).

We analysed gene expression variations to estimate the impact of using genetically unmatched cells. Variability between cell lines was greater than within cell lines for each genotype (1.7 ± 0.4 times on average for healthy cells and 3.9 ± 0.7 times for DMD cells, *Figure* S3A). Variability within each cell line was equivalent in healthy and DMD cells (*Figure* S3B), while variability between healthy cell lines was lower than between DMD cell lines (*Figure* S3C). *Figure* S4 gives detailed gene expression of *SOX2*, *SOX5*, *PAX3*, *SGCA*, and *MYH3* to illustrate gene expression variations between cell differentiation and between cell lines.

### Duchenne muscular dystrophy is initiated prior to the expression of skeletal muscle markers

First, the expression profile of the *DMD* variants was studied by RT‐qPCR in healthy and DMD hiPSCs during the differentiation process described in *Figure* S2A. The *Dp427m* variant, which is normally observed in muscle cells,^41^ appeared from Day 3 and was increased at Day 17, in contrast with *Dp412e*—the embryonic variant of dystrophin present in mesoderm cells^15^—which was expressed from Day 0, increased at differentiation Day 3, and disappeared from Day 10. Therefore, the expression of the *DMD* locus is initiated in the very first steps of the differentiation protocol, well before the entry into the skeletal muscle lineage. The ubiquitous variant *Dp71‐40* was detected at every time point, in contrast with *Dp116* (Schwann cell variant^42^), *Dp140* (kidney and foetal brain variant^43^) *Dp427p1p2* (Purkinje cell variant^44^), and *Dp427c*, which were either undetected or expressed at very low levels (*Figure* S2B). Interestingly, *Dp260* (retinal variant^45^) followed a similar expression pattern than *Dp427m*.

**Figure 1:**
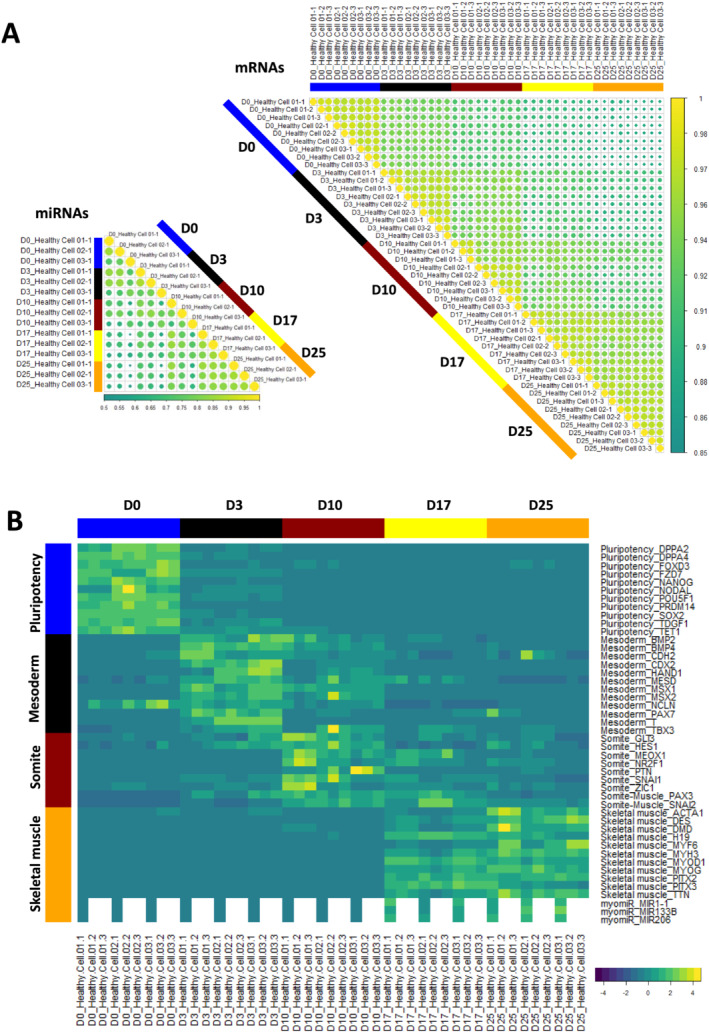
Differentiation dynamics of hiPSCs (D0) into MyoT (D25) in healthy cells at the transcriptomic level. **(A)** Spearman correlation matrix of transcriptomes (mRNAs, right) and miRnomes (miRNAs, left). Yellow dots indicate a stronger correlation. **(B)** Gene expression heatmap of selected differentiation markers (D: day; hiPSC: human induced pluripotent stem cell; MyoT: myotube).

A strong correlation in the transcriptomic data was observed by mRNA‐seq and miRNA‐seq between samples collected at an individual time point, as opposed to samples from two distinct time points. In addition, the correlation coefficient between samples taken at two successive time points increased as differentiation progressed (*Figure* 1A). Differential expression analysis in healthy controls between two successive collection days (Days 3/0, Days 10/3, Days 17/10, Days 25/17) showed that the proportion of regulated genes decreased from 26% to 18% of the whole transcriptome through the course of differentiation (8080 to 5320 mRNAs, adjusted *P* value ≤ 0.01, *Figure* S1A). These observations demonstrate the robustness of the differentiation protocol and are in agreement with an early specialization and a later refinement of the transcriptome as cells quickly exit pluripotency and become progressively restricted to the skeletal muscle lineage.

**Figure 2:**
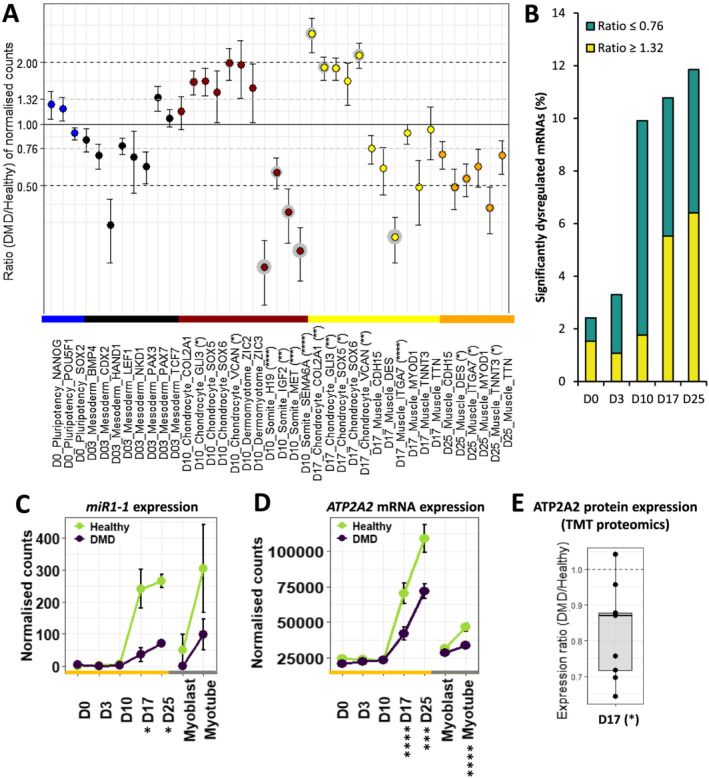
Differentiation dynamics of hiPSCs (D0) into MyoT (D25) in Duchenne muscular dystrophy (DMD) cells. **(A)** Dotplot of DMD/healthy expression ratios of selected differentiation markers. Statistical differences are indicated in brackets after gene names and grey circles around the corresponding dots. **(B)** Proportions of significantly dysregulated mRNAs (adjusted *P* value ≤ 0.05) in DMD cells at each time point. Expression of **(C)** *MIR1–1* and **(D)** *ATP2A2* mRNA during differentiation. (E) ATP2A2 protein level at D17 (*adjusted *P* value ≤ 0.05, **adjusted *P* value ≤ 0.01, ***adjusted *P* value ≤ 0.001, ****adjusted *P* value ≤ 0.0001; D: day; hiPSC: human induced pluripotent stem cell; MyoT: myotube).

To characterize the developmental stages achieved by the cells, the expression of lineage‐specific markers (both mRNAs and miRNAs) was determined at each time point, together with gene ontology enrichment analyses (*Figures* 1B, 2A, S1B, and S1C; *Table* S5).

Pluripotency was similarly maintained in healthy and DMD cells at Day 0 (*Figure* 2A, *Table* S5), as already shown by our group.^15^ At Day 3, cells lost pluripotency and became paraxial mesoderm cells expressing marker genes such as *PAX3* and *PAX7* (*Figures* 2A and S5A, *Table* S5). Importantly, markers of lateral plate (e.g. *GATA4*) and intermediate mesoderm (e.g. *PAX8*) were not up‐regulated at this stage (*Table* S5). Similarly, earlier markers of primitive streak (e.g. *TBX6*) and mesendoderm (e.g. *MIXL1*), as well as markers of the other germ layers, endoderm (e.g. *SOX17*) and ectoderm (e.g. *SOX2*), were either not expressed, greatly down‐regulated, or expressed at very low levels (*Table* S5), suggesting cell homogeneity in the differentiation process. Pluripotency markers as well as transgenes were properly down‐regulated/silenced all along cell differentiation (*Figure* S5)—except for *MYC* at the later stages in one healthy cell line. However, the transgene partial reactivation did not have a quantifiable impact neither on total *MYC* expression (*Figure* S5B) nor on global transcriptomics data, as shown by Spearman correlation coefficients between the three healthy lines at D17 and D25 (*Figure* S5D).

At that early time point, DMD‐associated gene dysregulation represented less than 3% of the entire transcriptome (adjusted *P* value ≤ 0.05, *Figure* 2B) but already contained genes important for development (e.g. *MEIS2*) and muscle formation (e.g. *ACTA1*). However, mesoderm markers were not significantly dysregulated, attesting that mesoderm commitment was mostly unimpaired (*Figure* 2A, *Table* S5). No increase in the expression of primitive streak, mesendoderm, endoderm, or ectoderm markers was detected, suggesting no differences in the differentiation process of DMD cells at that stage (*Table* S5).

In contrast, a sharp increase in the proportion of dysregulated genes appeared at Day 10, mostly including gene down‐regulations (DMD/healthy expression ratio ≤ 0.76, adjusted *P* value ≤ 0.05). This concerned almost 10% of the transcriptome at Day 10 (against 3% at Day 3) and remained stable from 10% to 12% (1226 mRNAs) until Day 25 (*Figure* 2B). At Day 10, healthy cells expressed genes typically observed during somitogenesis, such as *PAX3*, *NR2F2*, *PTN*, *MET*, *H19*, and *IGF2* (*Table* S5). More precisely, their transcriptome exhibits a mixed profile between dermomyotome (expression of *GLI3* and *GAS1* but not *ZIC3*) and myotome (expression of *MET* and *EPHA4* but not *LBX1*) (*Table* S5). Neither markers of presomitic mesoderm cells (e.g. *FGF8*) and neural plate cells (e.g. *FOXD3*) nor markers of sclerotome (e.g. *PAX1*) and dermatome (e.g. *EGFL6*) were up‐regulated (*Table* S5) in both healthy and DMD cells. In the meantime, several somite markers were down‐regulated, including *H19*, *IGF2*, *MET*, and *SEMA6A* (validated at the protein level for SEMA6A, *Figures* 2A and S6A, *Table* S5), while a slight up‐regulation of chondrocyte markers was highlighted and confirmed at the protein level for GLI3 (*Figure* S6B), together with a significant enrichment of the gene ontology term ‘nervous system development’, suggesting potential lineage bifurcations at Day 10 (*Figures* 2A and S1C, *Table* S5).

The study of differentiation dynamics presented above highlights that mesoderm commitment is not impaired by the absence of dystrophin and shows that DMD onset takes place at the somite cell stage, before the expression of the skeletal muscle program and especially before the up‐regulation of *Dp427m* expression.

### Duchenne muscular dystrophy skeletal muscle progenitor cells exhibit specific muscle gene dysregulations

Healthy and DMD cells were in the skeletal muscle compartment at Day 17, as evidenced by the expression of multiple lineage‐specific genes, such as transcription factors (e.g. *MYOD1*), cell surface markers (e.g. *CDH15*), sarcomere genes (e.g. *TNNC2*), dystrophin‐associated protein complex (DAPC) genes (e.g. *SGCA*), calcium homeostasis genes (e.g. *RYR1*), and muscle‐specific miRNAs (myomiR, e.g. *MIR1‐1*, *MIR206*, and *MIR133*). This was also observed at the protein level for CDH15, TNNC2, and RYR1 (*Figure* 1B, *Table* S5). They both showed an embryonic/foetal phenotype characterized by *ERBB3* expression, in contrast with tissue‐derived myoblasts that expressed *NGFR*. *PAX7* and *CD34*, two markers of skeletal muscle stem cells, were 12 and 23 times less expressed in hiPSC‐derived cells than in their tissue‐derived myoblasts (*Figure* S7, *Table* S5). Here again, alternative cell lineages were absent or greatly down‐regulated, such as tenocytes (e.g. *MKX*), chondrocytes (e.g. *SOX5*), osteoblasts (e.g. *SPP1*), or nephron progenitors (e.g. *SALL1*) (*Table* S5). Purity of the cell cultures was confirmed by single‐cell RNA‐seq in one healthy cell line (*Figure* S8). For instance, expression of *SOX2* (a reprogramming factor) and *OLIG2* (a moto‐neuron marker) was barely detected, while a large majority of cells expressed the skeletal muscle markers *MYOD1* and *ACTC1*.

Interestingly, DMD cells did not show a significant dysregulation of skeletal muscle transcription factors (*Table* S5). However, several myomiRs were found down‐regulated (e.g. *MIR1‐1*, *Figure* 2C), together with genes related to calcium homeostasis (e.g. *ATP2A2*, at both mRNA and protein level, *Figure* 2D and 2E) as well as members of the DAPC (e.g. *SNTA1*) (*Table* S5). Concerning cell lineages, there was no visible difference when compared with healthy controls, except an up‐regulation of markers associated with chondrocytes, which was confirmed at the protein level for GLI3 (*Figure* S6C), and a significant enrichment of the gene ontology term ‘nervous system development’ previously seen at Day 10, together with ‘kidney development’ and ‘ossification’ (*Figures* 2A and S1C, *Table* S5). To further consolidate the findings from hiPSC‐derived cells, a pair of isogenic hESCs was differentiated up to the myotube stage. Like in the DMD hiPSC‐derived cells, *DMD* was down‐regulated in the DMD Delta52 hESC‐derived cells (*Figure* S9A). The absence of dystrophin was confirmed by western blot (*Figure* S9B). Dysregulations of a selection of 10 genes were similar to those observed with the hiPSC model (Spearman correlation of *r* = 0.89, *P* value = 0.0012, *Figure* S9C).

Duchenne muscular dystrophy‐specific dysregulations were further queried at the protein level using TMT proteomics. A total of 3826 proteins were detected in the six processed samples (three healthy and three DMD, *Table* S3). Among this list, 185 proteins (139 + 46) were found significantly dysregulated in DMD and 375 (329 + 46) of the corresponding mRNAs were previously detected dysregulated in the RNA‐seq analysis, the overlap between protein and mRNA identified dysregulations being 46 (|log2FoldChange| ≥ 0.4 and adjusted *P* value ≤ 0.05, *Figure* S6D and S6E, *Table* S6). Moreover, among the total of 514 genes represented in *Figure* S6F, 98 were dysregulated alike in both datasets (56 up‐regulated + 42 down‐regulated) against 13 (12 + 1) in the opposite direction (|log2FoldChange| ≥ 0.4, *Figure* S6F, *Table* S6) resulting in a Spearman correlation of *r* = 0.49 and *P* value < 0.0001. In this mRNA/protein comparison, the mRNA experiment was more sensitive than protein experiment and could also be considered as a good proxy for proteins.

To better characterize the most direct consequences of the loss of *DMD* in muscle cells, *DMD* expression was either knocked down or rescued at Day 17 by transient exon skipping using a specific PMO targeting *DMD* exon 7 or 51. Treatment with PMO7 in a healthy hiPSC line resulted in significant exon skipping, which was correlated with reduced *DMD* expression up to 94% (Spearman *r* = −0.88, analysed pairs = 59, *P* value < 0.0001, *Figure* S10A, S10D, and S10E) and reduced dystrophin protein levels (up to 81%, *Figure* S10C). In parallel, treatment with PMO51 in DMD Delta52 hESC‐derived cells also resulted in significant exon skipping (100% and 92.4%, respectively, with the 100 μM PMO concentration, *Figure* S10B). This was correlated with an increased expression of *DMD* (Spearman *r* = 0.65, analysed pairs = 27, *P* value = 0.0002 for the DMD hiPSC line; *r* = 0.89, analysed pairs = 20, *P* value < 0.0001 for the DMD Delta52 hESC‐derived cells, *Figure* S10D and S10E) and the expression of the dystrophin protein (*Figure* S10C). The expression of specific transcripts was measured by RT‐qPCR the 3 following days (*Figure* S10D): expression of *MYH3*, *MYOG*, and *SGCA* was significantly affected by PMO treatment, while transcripts coding for *DES* and *ITGA7* were not.

Therefore, DMD cells efficiently enter the skeletal muscle compartment at Day 17 but exhibit dysregulations in several features typically associated with dystrophic muscles, which could be a consequence of the early manifestations of DMD detected at Day 10. Some of these identified dysregulations were mimicked by transient *DMD* knockdown and partly abolished by transient *DMD* rescue.

### Human induced pluripotent stem cell differentiation leads to embryonic/foetal myotubes that reproduce Duchenne muscular dystrophy phenotypes

**Figure 3:**
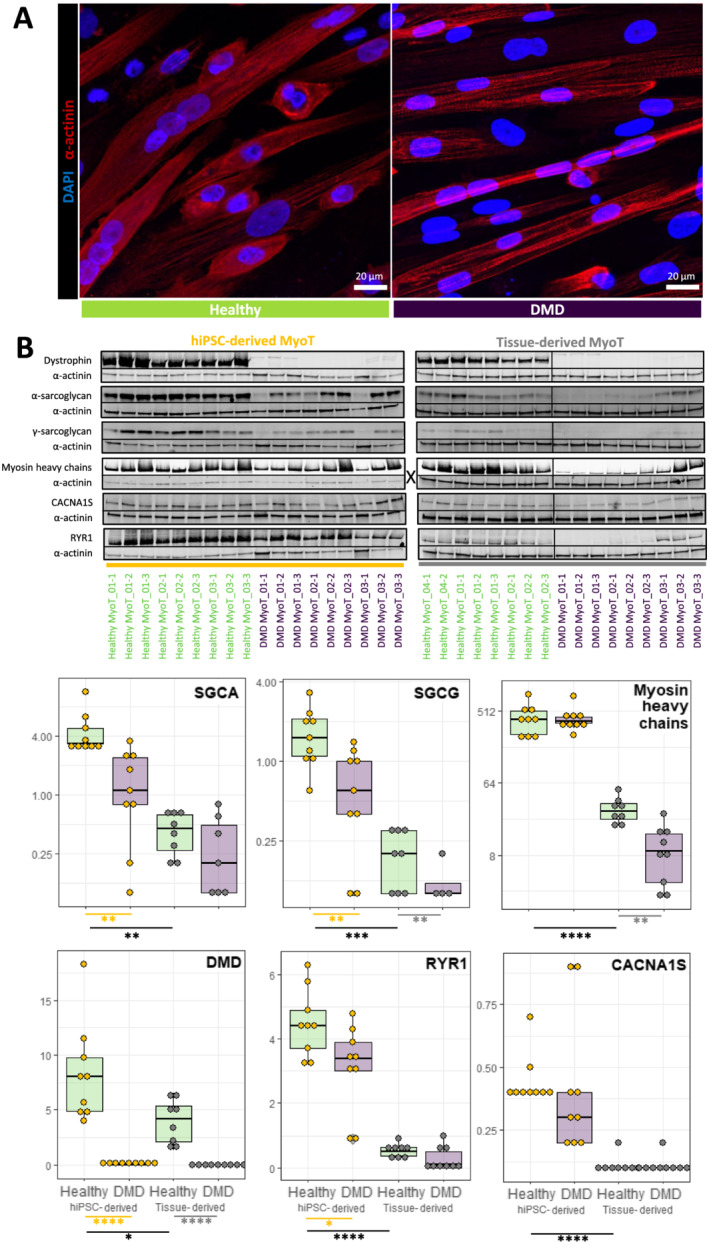
Comparison of healthy and Duchenne muscular dystrophy (DMD) MyoT from hiPSCs and tissues at the protein level. **(A)** hiPSC‐derived MyoT immunolabelling of α‐actinin (red) and nuclei (DAPI, blue) in healthy (left) and DMD cells (right). **(B)** Representative western blots and related quantifications of DMD, SGCA, SGCG, myosin heavy chains, CACNA1S, and RYR1 from protein extracts in healthy and DMD hiPSC‐derived and tissue‐derived MyoT (X: 0.25 μg of total protein was used in hiPSC‐derived MyoT instead of 7 μg in tissue‐derived MyoT—*P value ≤ 0.05, **P value ≤ 0.01, ***P value ≤ 0.001, ****P value ≤ 0.0001) (hiPSC: human induced pluripotent stem cell; MyoT: myotube).

As previously described,^23^ both healthy and DMD hiPSC‐derived myotubes (Day 25) were able to twitch spontaneously in culture, and fluorescent staining of nuclei and α‐actinin confirmed cell fusion and the formation of striation patterns typical of muscle fibres *in vivo* (*Figure* 3A). Western blot analyses on protein extracts from DMD cells confirmed that dystrophin was either undetectable or slightly expressed (*Figure* 3B), as in the corresponding patient muscle biopsies (data not shown).

**Figure 4:**
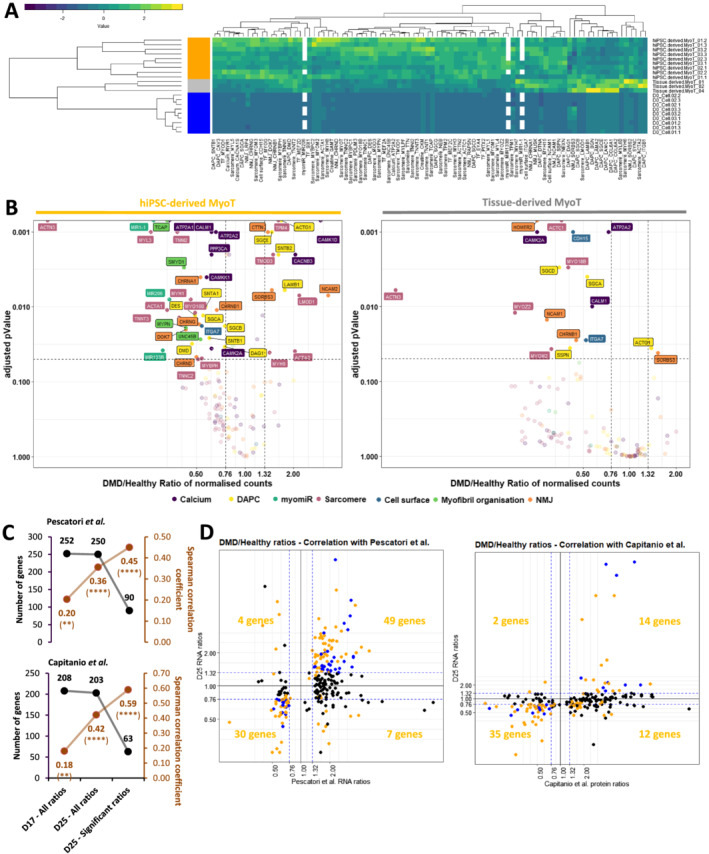
Manifestation of the Duchenne muscular dystrophy (DMD) phenotype in the transcriptome and miRnome of myotubes derived from hiPSCs and tissues. **(A)** Hierarchical clustering and heatmap in healthy hiPSCs (D0), hiPSC‐derived MyoT, and tissue‐derived MyoT with selected skeletal muscle transcripts and miRNAs. **(B)** Volcano plots of dysregulated mRNAs/miRNAs in hiPSC‐derived MyoT (left) and tissue‐derived MyoT (right)—vertical grey dashed lines represent DMD/healthy ratio thresholds at 0.76 or 1.32, and the horizontal grey dashed line represents the adjusted *P* value threshold at 0.05. Comparisons of DMD/healthy expression ratios at D17 and D25 with published omics data from muscle biopsies^4^, ^46^: **(C)** number of genes in black and Spearman correlation coefficients in brown found in common with Pescatori *et al*.'s mRNA data (top) and Capitanio *et al*.'s protein data (bottom) as well as **(D)** correlation graphs of the D25 data compared with Pescatori *et al*.'s mRNA data (left) and Capitanio *et al*.'s protein data (right). Genes with |log2FoldChange| ≥ 0.4 are in blue if adjusted *P* value ≥ 0.05 and yellow if adjusted *P* value ≤ 0.05 (DAPC: dystrophin‐associated protein complex; hiPSC: human induced pluripotent stem cell; MyoT: myotube; NMJ: neuromuscular junction; TF: transcription factor MyoT—*P value ≤ 0.05, **P value ≤ 0.01, ***P value ≤ 0.001, ****P value ≤ 0.0001).

We selected representative mRNAs and miRNAs and showed that both hiPSC‐derived and tissue‐derived myotubes have exited the cell cycle and up‐regulated genes expressed in skeletal muscles (*Figures* S4A and S12A, *Table* S5). This included skeletal muscle myomiRs (*MIR1‐1*, *MIR133*, and *MIR206*), transcription factors involved in skeletal myogenesis including those of the muscle regulatory factor family (e.g. *MYOD*, *MYOG*), and specific muscle cell surface markers (e.g. *CDH15*, *ITGA7*), as well as genes involved in the formation of the DAPC (e.g. *SGCA*, *DTNA*), sarcomeres (e.g. *TNNC2*, *TNNT3*), myofibril organization (e.g. *UNC45B*, *NACA*), and the triggering of excitation–contraction coupling at the neuromuscular junction (NMJ, e.g. *MUSK*, *DOK7*) (*Figure* 4A, *Table* S5). Neither visual nor transcriptional cell death was noticed in DMD myotubes (*Figure* S11).

Even though global analysis showed that hiPSC‐derived myotubes were similar to their tissue‐derived counterparts in term of lineage commitment, they displayed an embryonic/foetal phenotype—as suggested in progenitors at Day 17. This can be illustrated by the expression of the embryonic/foetal myosin heavy/light chains *MYH3*, *MYH8*, *MYL4*, and *MYL5* but not the postnatal transcripts *MYH1* and *MYH2*, which were detected in tissue‐derived myotubes. Myotubes derived from hiPSCs had also higher levels of *IGF2*, which is down‐regulated at birth,^47^ and expressed *DLK1*, which is known to be extinct in adult muscles^48^ (*Figure* S12B).

Despite the embryonic/foetal phenotype, hiPSC‐derived myotubes showed evidence of terminal differentiation and cellular maturation. First, their total level of myosin heavy chain proteins was significantly higher than in tissue‐derived myotubes, as confirmed by western blotting (*Figure* 3B). RNAs and proteins involved in DAPC formation (e.g. DMD, SGCA, and SGCG), as well as in excitation–contraction coupling (e.g. RYR1 and CACNA1S/CAV1.1), were also present at higher levels (*Figures* 3B and 4A). Finally, higher expression of skeletal muscle transcription factors (e.g. *MEF2C*) and of multiple genes involved in muscle contraction (e.g. *TNNT3*), NMJ formation (e.g. *RAPSN*), and creatine metabolism (e.g. *CKM*) indicates that hiPSC‐derived cells expressed features of fully differentiated muscle cells (*Figure* 4A). Similar to previous time points, Day 25 cells were negative for markers of alternative muscle lineages, that is, cardiac (*MIR208a*, *MYL7*, and *RYR2*) and smooth muscle cells (*MYH11*, *CNN1*, and *CHRNA3/B2/B4*) (*Table* S5).

In DMD cells, unbiased mRNA‐seq analysis highlighted striking transcriptome dysregulations with 3578 differentially expressed genes in hiPSC‐derived myotubes including well‐known muscle genes. There was a global trend towards down‐regulation of muscle transcription factors, which was only significant for *MEF2A* and *MEF2D* in hiPSC‐derived myotubes and *EYA4* and *MYOD1* in tissue‐derived myotubes (*Figure* S12C). In addition, myomiRs previously associated with muscle dystrophy (dystromiRs, e.g. *MIR1‐1*, *MIR206*, *and MIR133*, *Figures* 2C and 4B) were found down‐regulated (*Table* S5). Similarly, a global down‐regulation phenotype was observed in both tissue‐derived and hiPSC‐derived DMD myotubes and concerned multiple mRNAs and/or proteins associated with known disease phenotypes, such as cell surface markers (e.g. *ITGA7*), DAPC organization (e.g. both SGCA mRNA and protein as well as SGCG protein), myofibril organization (e.g. *UNC45B*), sarcomere formation (e.g. *MYO18B*), NMJ function (e.g. *CHRNB1*), and calcium homeostasis (e.g. *ATP2A2* mRNA and RYR1 protein) (*Figure* 3B for protein data, *Figure* 4B for transcript data, and *Figure* S1C for enrichment data). Like at Day 17, dysregulations of a selection of 10 genes were similar in the DMD Delta52 hESC‐derived cells and in the hiPSC model (Spearman correlation of *r* = 0.92, *P* value = 0.0004, *Figure* S9C).

Then, we compared the DMD/healthy expression ratios at Day 25 to two sets of published omics data from healthy and DMD muscle biopsies: one obtained at the mRNA level in pre‐symptomatic DMD patients younger than 2 years old^4^ and another at the protein level in patients aged from 9 months to 8 years old.^46^ Both datasets were closer to Day 25 cells (hiPSC‐derived myotubes) than Day 17 cells as expected. Our hiPSC‐derived myotubes expressed 250 of the 261 dysregulated genes and 203 of the 226 dysregulated proteins found in these respective studies (Spearman correlations of *r* = 0.36 and *r* = 0.42, *P* value < 0.0001, *Figure* 4C, *Table* S6). Among these, respectively 90 and 63 genes were also significantly dysregulated in our dataset (|log2FoldChange| ≥ 0.4, adjusted *P* value ≤ 0.05): 88% (79/90 genes) of the identified genes from the mRNA dataset and 78% (49/63 genes) of the identified genes from the protein dataset were dysregulated in the same direction, resulting in Spearman correlation of *r* = 0.45 and *r* = 0.59, respectively (*P* value ≤ 0.0001, *Figure* 4C and 4D, *Table* S6).

Altogether, these data indicate that hiPSC‐derived myotubes recapitulate a full skeletal muscle differentiation program and exhibit an embryonic/foetal phenotype. Despite that, it shows that DMD phenotypes are already detectable at the transcriptional level and correlated with those found in human patients. This validates the relevance of this cell system to model the DMD pathology.

### Markers of fibrosis are intrinsic to Duchenne muscular dystrophy human induced pluripotent stem cell‐derived myotubes

**Figure 5:**
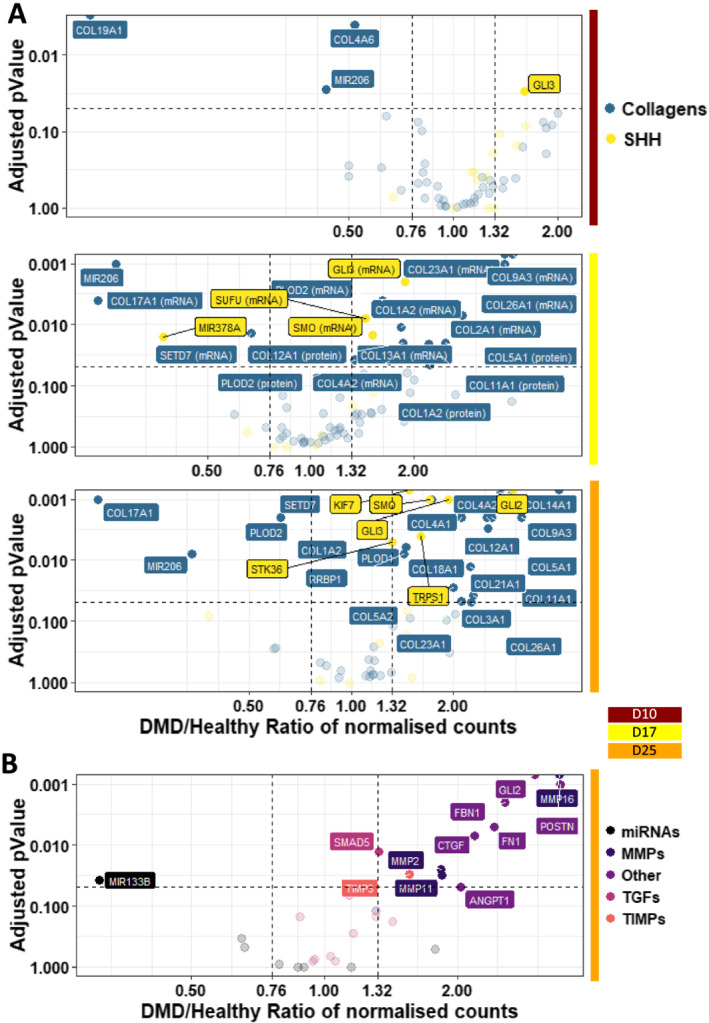
Illustration of the fibrosis phenotypes in Duchenne muscular dystrophy (DMD) cells. Volcano plots of dysregulated mRNAs/miRNAs related to (A) the SHH pathway and collagen metabolism at D10/17/25 and (B) fibrosis at D25—vertical grey dashed lines represent DMD/healthy ratio thresholds at 0.76 or 1.32, and the horizontal grey dashed line represents the adjusted *P* value threshold at 0.05 (D: day; MMP: matrix metallopeptidase; SHH: sonic hedgehog pathway; TIMP: tissue inhibitor of metallopeptidase; TGF: transforming growth factor).

As presented above, the up‐regulation of chondrocyte markers in DMD cells, although already present at Day 10, became significant from Day 17 (*Figure* 2A, *Table* S5). It was accompanied by the up‐regulations of the SHH signalling pathway and of multiple collagens (*Figure* 5A, *Table* S5). Genes encoding the *P4H* collagen synthases were not dysregulated, while *RRBP1* (that stimulates collagen synthesis) together with *PLOD1* and *PLOD2* (that stabilize collagens) were significantly up‐regulated. Moreover, *SETD7*, a gene known for activating collagenases, was significantly down‐regulated.

At the myotube stage, a fibrosis‐related gene set was clearly up‐regulated in DMD cells, as illustrated by the overexpression of *ANGPT1*, *CTGF*, collagens (e.g. *COL1A2*), matrix metallopeptidases (*MMPs*), and tissue inhibitors of metallopeptidase (*TIMPs*) (*Figure* 5B). Conversely, the myomiR *MIR133* that controls *CTGF* expression^49^ was repressed (*Table* S5). Interestingly, gene members of the transforming growth factor (TGF)‐β pathway, a well‐known inducer of fibrosis,^50^ were not found dysregulated (*Figure* 5B, *Table* S5).

Altogether, these data argue for fibrosis as an intrinsic feature of DMD skeletal muscle cells, rather than a process solely driven by interstitial cell populations in the niche. Furthermore, this muscle‐driven fibrosis seems independent of the TGF‐β pathway, and could rather depend on the SHH pathway, together with an intrinsic up‐regulation of chondrocyte markers and collagens.

### Genes involved in mitochondrial metabolism are drastically dysregulated in Duchenne muscular dystrophy human induced pluripotent stem cell‐derived myotubes

**Figure 6:**
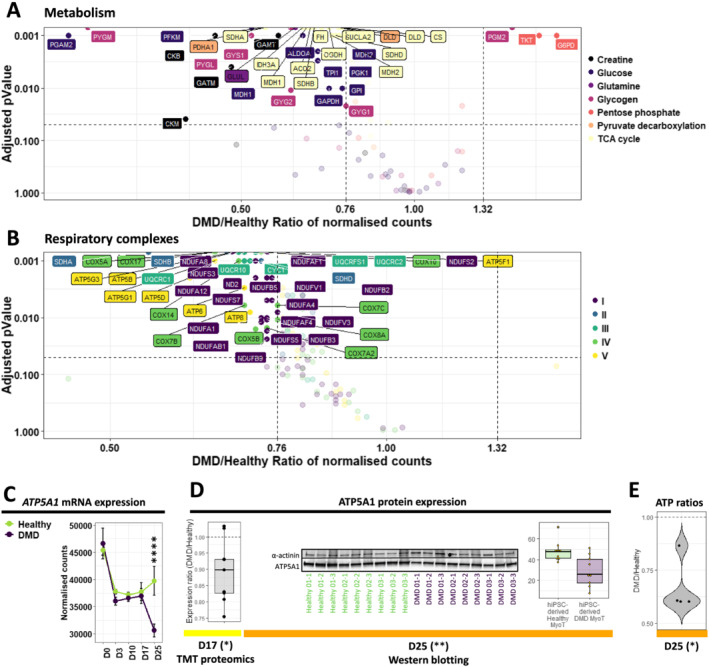
Illustration of the metabolic and mitochondrial phenotypes in Duchenne muscular dystrophy (DMD) cells. Volcano plots of dysregulated mRNAs/miRNAs related to (A) principal metabolic pathways and (B) the constitution of the five mitochondrial respiratory complexes in DMD hiPSC‐derived MyoT—vertical grey dashed lines represent DMD/healthy ratio thresholds at 0.76 or 1.32, and the horizontal grey dashed line represents the adjusted *P* value threshold at 0.05. Quantification of ATP5A1 expression (C) at the mRNA level during differentiation and (D) at the protein level at D17 (TMT proteomic data, left) and D25 (western blot data, right). (E) Measure of ATP levels in DMD cell lines, relative to healthy controls (*adjusted *P* value ≤ 0.05, **adjusted *P* value ≤ 0.01, ***adjusted *P* value ≤ 0.001, ****adjusted *P* value ≤ 0.0001) (D: day; hiPSC: human induced pluripotent stem cell, MyoT: myotube)

As previously described^51^ and illustrated on *Figure* S13A, genes involved in the energy metabolism of DMD hiPSC‐derived myotubes were dysregulated at the creatine and carbohydrate levels, up to the respiration (*Figures* 6A, 6B, and S1C; *Table* S5). The creatine transporter was not impacted, while mRNAs coding for enzymes of both creatine and creatine phosphate biosynthesis were underrepresented. Neither glucose nor glutamate transporter expression were impaired. However, genes involved in glutamine biosynthesis (followed by gluconeogenesis that feeds glycolysis from glutamine) as well as glycogenesis (followed by glycogenolysis that feeds glycolysis from glycogen) were all down‐regulated, together with genes coding for glycolysis itself. In contrast, genes coding for the pentose phosphate pathway (which is in parallel to glycolysis) were up‐regulated, especially the oxidative part. Gene expression for pyruvate decarboxylation and generation of acetyl‐CoA to feed the tricarboxylic acid cycle was also impaired. Finally, the genes involved in the tricarboxylic acid cycle itself (*Figure*s 6A and S1C) and the mitochondrial electron transport chain were down‐regulated (*Figures* 6B and S1C). This is particularly reinforced by lower levels of a member of the ATP synthase complex ATP5A1 at both mRNA and protein levels (*Figure* 6C and 6D). These mRNA and protein data were completed by the measurement of ATP levels, which were significantly decreased in DMD hiPSC‐derived myotubes (*Figure* 6E). Moreover, transcripts encoded by the mitochondrial DNA and mitochondrial DNA itself were decreased in DMD hiPSC‐derived myotubes at Day 25 (*Figure* S13B–E).

**Figure 7:**
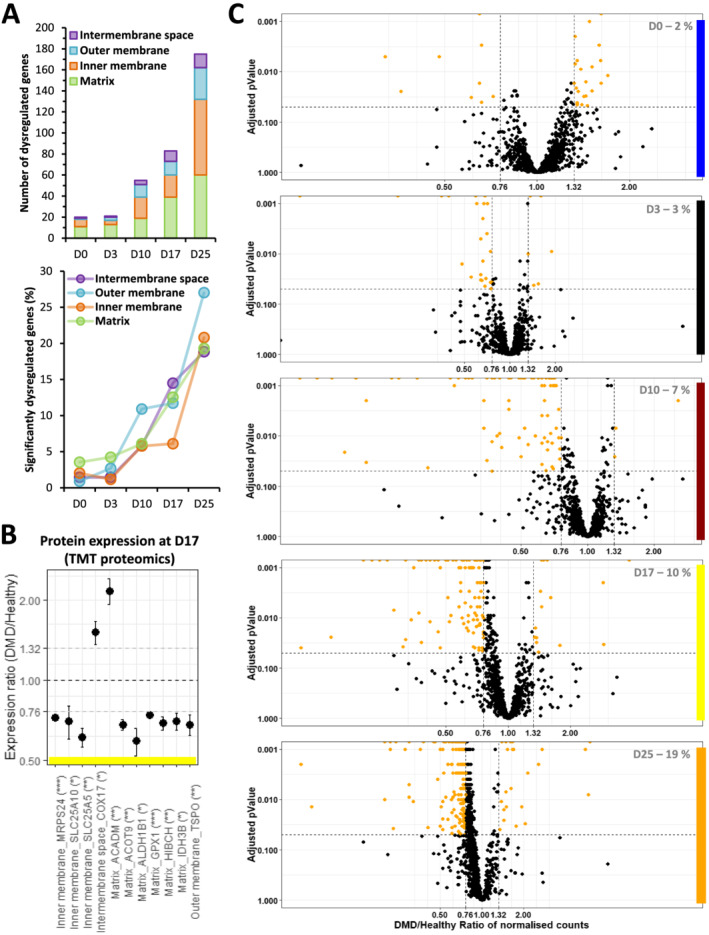
Mitochondrial dysregulations in Duchenne muscular dystrophy (DMD) cells during differentiation. (A) Absolute (top) and relative numbers (%, bottom) of dysregulated genes from the different mitochondrial compartments over the course of DMD hiPSC differentiation. (B) Expression ratios of selected mitochondrial proteins. Statistical differences are indicated in brackets (*adjusted *P* value ≤ 0.05, **adjusted *P* value ≤ 0.01, ***adjusted *P* value ≤ 0.001, ****adjusted *P* value ≤ 0.0001). (C) Volcano plots of mitochondria‐related genes over the course of DMD hiPSC differentiation. Statistical differences are symbolized with orange dots—vertical grey dashed lines represent DMD/healthy ratio thresholds at 0.76 or 1.32, and the horizontal grey dashed line represents the adjusted *P* value threshold at 0.05. The percentage of significantly dysregulated genes is indicated at the bottom right in grey (D: day).

In the presented cell model, a significant down‐regulation of an mRNA set coding for mitochondrial proteins was primarily observed at Day 10 with the down‐regulation of 11% (12 mRNAs, DMD/healthy expression ratio ≤ 0.76, adjusted *P* value ≤ 0.05) of the mitochondrial outer membrane genes and amplified during the differentiation of DMD cells (*Figure* 7A). Therefore, defects depicted at Day 25 rooted before the expression of the skeletal muscle program at Day 17. Among them, mRNA down‐regulation of *TSPO*, a channel‐like molecule involved in the modulation of mitochondrial transition pore,^52^ occurred from Day 10 to Day 25. This down‐regulation was also observed at the protein level at Day 17 (*Figure* 7B). Moreover, the protein import system was affected from Day 17 at both mRNA and protein levels (*Figure* S13C–F). Simultaneously, mRNAs involved in mitochondrial genome transcription started to be down‐regulated, followed by genes involved in mitochondrial DNA replication at Day 25 (*Figure* S13D–G). This progressive increase of dysregulations was also observed at the level of the entire mRNA set related to mitochondria (around 1000 mRNAs) as illustrated by the volcano plots as well as the gene ontology enrichments (*Figures* 7C and S1C).

Our data highlight early impairments in genes coding for mitochondria that start at the somite stage and increase with the differentiation in an orderly manner. These elements complete the mitochondrial DMD phenotype described above at the myotube stage.

Altogether, our study demonstrates that DMD starts prior to the expression of well‐described markers of muscle differentiation. It shows that hiPSC‐based experimental models of DMD can help identify early disease manifestations and stratify multiple pathological features over the course of muscle development.

Additional references describing the expression of marker genes at specific differentiation steps can be found in the Data S1.

## Discussion

Since the discovery of the *DMD* gene in 1987,^1^ DMD cellular phenotypes were considered under the unique scope of a ‘mechanical hypothesis’ in which dystrophin deficiency led to membrane leakage and ultimately muscle cell rupture. However, over the last 15–20 years, studies have brought unequivocal evidence that multiple additional factors are in play, such as calcium intracellular overloads,^53^, ^54^ excessive oxidative stress,^55^, ^56^ metabolic switches,^57^, ^58^ as well as an overall tissue context where aberrant interactions between resident cells lead to inflammation and fibro‐adipogenesis.^59^, ^60^, ^61^ This has progressively led to a complex picture involving interdependent homeostatic perturbations, and to date, the identification of prevalent pathological features driving the initiation of DMD is hardly feasible.

The skeletal myogenesis modelled here by the differentiation of hiPSCs, without gene overexpression or cell sorting, homogeneously and robustly recapitulates key developmental steps—pluripotency, mesoderm, somite, and skeletal muscle—without any trace of other lineages. As shown by the analysis of gene expression variations between triplicates, variability between cell lines was greater than within cell lines for each genotype. The different genetic backgrounds can explain this. In the meantime, healthy cells exhibited less variability between each other than DMD cells that bear distinct *DMD* mutations. We can therefore argue that our phenotypical analysis will have more false negatives than false positives. It can also be considered closer to clinical heterogeneity. Therefore, it is a suitable dynamic model for studying human skeletal muscle development in both healthy and DMD cells, offering the possibility to clarify the consequences of the absence of dystrophin at each step of the differentiation process, as well as to explore dystrophin functions and find earlier and more specific disease biomarkers.

As previously observed with pluripotent stem cells,^62^ hiPSC‐derived myotubes at Day 25 displayed an embryonic/foetal gene expression profile. However, a clear distinction must be made between the nature of the expressed isoforms—embryonic/foetal/postnatal—and the degree of differentiation. For instance, hiPSC‐derived myotubes expressed multiple markers of terminally differentiated muscles at levels higher than those measured in tissue‐derived myotubes. With the idea of exploring human DMD phenotypes during muscle development, we argued that generating embryonic/foetal myotubes from hiPSCs would not be a limitation.

In qualitative terms, DMD hiPSC‐derived myotubes showed an overall morphology similar to healthy controls, with cell fusion and clear striation patterns, suggesting that the potential impact of dystrophin during *in vitro* differentiation is subtle and does not prevent myotube formation. However, our unbiased mRNA‐seq analysis highlighted striking transcriptome dysregulations at Day 25. This includes numerous genes that can be linked to previously described DMD phenotypes such as (i) DAPC dissociation,^63^ (ii) rupture of calcium homeostasis,^53^ (iii) myomiR down‐regulation,^64^, ^65^ (iv) sarcomere destabilization,^66^, ^67^ (v) mitochondrial and metabolism dysregulations,^57^, ^58^ (vi) NMJ fragmentation,^68^, ^69^ and (vii) fibrosis.^61^, ^70^ It is interesting to note that these phenotypes are already detected at the transcriptional level in embryonic/foetal myotubes, while they usually appear postnatally in human patients and other animal models. In addition, most of them are often considered as consequences of degeneration–regeneration cycles typical of DMD muscles *in vivo*,^71^, ^72^, ^73^ which are absent in our *in vitro* model, indicating that a part of these defects is primarily due to the absence of dystrophin itself. In particular, our data suggest that fibrosis is an intrinsic feature of DMD skeletal muscle cells, and therefore, it does not absolutely require a specific tissue context or additional cell populations to be detected *in vitro*. Fibrosis is a major hallmark of DMD pathophysiology, and the regulation of this process has been largely investigated in the past.^50^, ^74^ A long‐debated question is the implication of the TGFβ signalling pathway.^75^, ^76^ In this study, TGFβ signalling was inhibited up to Day 17 by specific molecules contained in the cell culture media, and TGFβ‐related genes were not up‐regulated at Day 25, suggesting that the observed up‐regulation of fibrosis‐related markers is TGFβ‐independent.

Because several studies in human patients and animal models had described dystrophic phenotypes in DMD foetuses/infants,^9^ ^–^ ^14^ we investigated the precise timing of disease onset in our hiPSC‐derived cells. First, the absence of dystrophin does not modify the capacity of cells derived from adult tissue biopsies to be reprogramed using the approach developed by Takahashi *et al*.^77^ Both healthy and DMD cells retained pluripotency and the capacity to enter the mesoderm compartment at Day 3. At that time, the embryonic dystrophin Dp412e is expressed and only marginal dysregulations are observed in DMD cells, *a priori* unrelated to cell fate choice as cells only express paraxial mesoderm markers at levels similar to healthy controls.

Duchenne muscular dystrophy dysregulations are greatly increased at Day 10, when cells express somite markers. At that time, we noticed few significant dysregulations of cell lineage markers, which became more prevalent at Days 17 and 25. This might be an indication that to some extent, cell fate is misguided in DMD cells, where skeletal muscle markers are underexpressed and replaced by markers of alternative lineages, such as chondrocytes.

First visible at Day 10, we identified the dysregulation of mitochondrial genes as one of the key processes happening in an orderly manner. Interestingly, early observations prior to the discovery of the *DMD* gene had hypothesized that DMD was a mitochondrial/metabolic disease based on protein quantifications and enzyme activities.^57^, ^78^ Later, mitochondria was identified as a key organelle in DMD, responsible for metabolic perturbations but also calcium accumulation and generation of reactive oxygen species.^53^ ^–^ ^56^ In this study, numerous genes coding for proteins located in the outer mitochondrial membrane start to be down‐regulated from Day 10 in DMD cells, such as the benzodiazepine receptor TSPO, a member of the controversial mitochondrial permeability transition pore (mPTP).^52^ The mPTP is a multiprotein complex whose members are not all precisely identified, and several studies suggest that it might be involved in DMD pathophysiology.^79^, ^80^ A chicken‐and‐egg question currently debated relates to the initiation of these homeostatic breakdowns, as positive feedbacks exist between mitochondria, oxidative stress, and calcium homeostasis dysregulations.^54^, ^55^ At the transcriptome level, dysregulations of genes controlling calcium homeostasis were detected after Day 10, suggesting that mitochondrial impairment starts early and has predominant consequences in DMD, as hypothesized by Timpani *et al*.^51^ Further experiments are needed to better evaluate the impact of mitochondrial dysregulations at the functional level.

Day 17 marks the entry into the skeletal muscle compartment with the expression of specific transcription factors, cell surface markers, myomiRs, as well as the increase of skeletal muscle variant of dystrophin (*Dp427m*). It also marks the initiation of the skeletal muscle gene dysregulations observed at the myotube stage (i.e. down‐regulation of genes involved in DAPC and calcium homeostasis). For instance, the up‐regulation of fibrosis‐related genes observed in DMD myotubes at Day 25 is already visible at Day 17, with the up‐regulation of the SHH pathway as well as collagen‐related genes. In this study, it is seen as an early indicator of DMD physiopathology, confirming previous observations in DMD infants, both transcriptionally^4^ and histologically.^81^, ^82^

Moreover, several myomiRs were found down‐regulated at Days 17 and 25 and seem to play a central part in multiple DMD phenotypes. Besides their role in myogenesis,^83^, ^84^ myomiRs can be involved in calcium homeostasis,^85^ metabolism and mitochondrial functions,^86^, ^87^ and fibrosis.^49^, ^88^ In particular, *MIR1‐1* and *MIR206* are known to target key genes such as *CACNA1C*,^85^ *CTGF*,^49^ *RRBP1*,^88^ several regulators of the pentose phosphate pathway,^86^ and even transcripts encoded by the mitochondrial genome.^87^ Even though the functional consequences of the multiple gene and myomiR dysregulations highlighted in this study are virtually impossible to anticipate, we believe that myomiRs can be key players in DMD physiopathology.

Previous studies in mice suggest early functional roles of muscular dystrophin in the development of striated muscles^89^, ^90^ and show that dystrophin participates in regulating satellite cell polarity, asymmetric division, and possibly commitment.^91^, ^92^, ^93^ Few other studies in DMD hiPSC‐derived myoblasts^18^ and in DMD human primary myoblasts^94^ argued that DMD starts before the expression of the muscular dystrophin protein. Our data suggest that *Dp427m* is actually expressed before muscle commitment but at a lower level. This fact might explain why disease phenotypes seem to be initiated at the somite stage. This early initiation could also be explained by the deficit in other dystrophin isoforms expressed before Day 10, such as Dp412e at Day 3,^15^ as well as by the decrease or loss of other RNA products expressed from the *DMD* locus, such as the ubiquitous isoform Dp71‐40 or long non‐coding RNAs.^95^ The lack of knowledge around these additional products from the *DMD* locus contrasts with the extensive amount of data on the structure and function of the main muscular isoform Dp427m whose most studied role is to stabilize muscle cell membrane during contraction.^96^ *DMD* knockdown results at Day 17 in a healthy cell line with partial mimicking of DMD phenotype could suggest a dynamic process in DMD: some dysregulations might not be reproduced by removing *DMD* after muscle commitment, highlighting the fact that absence of DMD locus expression during development could have impacts before cells becoming muscles and therefore before Dp427m having its well‐known role in muscles, as it is shown by our multi‐omic study. The role of *Dp427m* in non‐muscle cells could also be questioned. Other tissue‐specific isoforms have been described, for example, in the retina (Dp260^45^) and in the brain (Dp427c,^97^ Dp427p,^44^ and Dp140^43^), some of which are also slightly expressed in skeletal muscles under certain circumstances,^98^ but their role remains mostly unknown. Interestingly, in our data, the expression of Dp260 follows the same pattern of expression as Dp427m. It has been shown that the expression of Dp260 in *mdx*/utrnK/K mice can rescue the *mdx* phenotype,^99^ indicating overlapping functions between Dp427m and Dp260. On the other hand, it is now well established that a third of DMD patients display cognitive deficiencies—which might be correlated with mutations affecting Dp140^100^—attesting that dystrophin can be involved in other cell functions.

To date, DMD diagnosis is made mostly around 4 years old when the first physical symptoms appear, meaning the muscles are already greatly damaged. Our study gives an argument for newborn screening. Moreover, the standard of care for DMD patients helps mitigate and delay some of the most severe symptoms but remains insufficient to have a curative effect. Despite decades of work with the *mdx* mouse model, only a few pharmacological candidate molecules have moved forward to clinical trials, with variable efficiency. As several gene therapy trials have been recently initiated with promising preliminary data, we believe that our human *in vitro* model system might be useful for the development of combination therapies. Recent studies have proved that the association of two different therapeutic approaches could have a synergistic effect on the overall treatment outcome and can be used for instance to boost the effect of dystrophin re‐expression by antisense oligonucleotides or gene therapy.^8^, ^101^, ^102^ Here, our extensive RNA‐seq data could help identify relevant therapeutic targets for pharmacological intervention, such as CTGF—involved in fibrosis and found up‐regulated in DMD myotubes—which can be inhibited by monoclonal antibodies,^103^ or TSPO receptor—a receptor potentially member of the mPTP down‐regulated in DMD cells—targetable with benzodiazepines.^104^ In addition, our model might also be used as a platform to screen pharmacological compounds in an unbiased high‐throughput manner. Indeed, skeletal muscle progenitor cells at Day 17 can be robustly amplified, cryopreserved, and plated in a 384‐well plate format (data not shown). Thus, they could be an interesting tool to highlight pharmacological compounds to be used alone or in combination with gene therapy.

To summarize, the directed differentiation of hiPSCs without gene overexpression or cell sorting homogeneously and robustly recapitulates key developmental steps of skeletal myogenesis and generates embryonic/foetal myotubes without any trace of other lineages. The absence of dystrophin does not compromise cell reprogramming, pluripotency, or the entry into the mesoderm compartment. While a very low amount of the long muscular dystrophin isoform is expressed, a significant transcriptome dysregulation can be observed at the somite stage that implicates mitochondria prior to dysregulations of genes controlling calcium homeostasis. Despite their ability to enter the skeletal muscle lineage compartment and become myotubes, DMD cells exhibit an imbalance in cell fate choice as they express lower amounts of key muscle proteins and retain basal expression of marker genes from other lineages, leading to the well‐characterized DMD phenotypes including muscle features and metabolism dysregulations as well as fibrosis. Altogether, these data argue for (i) a deficit and not a delay in DMD differentiation, (ii) seeing DMD as a progressive developmental disease as well as a metabolic pathology whose onset is triggered before the entry into the skeletal muscle compartment, and (iii) fibrosis as an intrinsic feature of DMD muscle cells. Future studies could explore the additional roles of *DMD* locus products and the impact of their loss during skeletal muscle development, as well as find earlier and more specific disease biomarkers and develop combination therapeutic strategies using high‐throughput drug screening.

### Data availability statement

All the omics data are available online for exploration through a graphical interface (https://muscle‐dmd.omics.ovh/). For additional information on this interface, please send an email to shiny@virginie‐mournetas.fr.

## Funding

We thank the Fondation Maladies Rares (GenOmics grant), Labex Revive (Investissement d'Avenir; ANR‐10‐LABX‐73), and the AFM Téléthon for funding this project.

## Conflicts of interest

Spiros D. Garbis is Founder, President and CEO of Proteas Bioanalytics, Inc.

## Supporting information


**Data S1.** Supporting Information

**Table S1.** Supporting Information

**Table S2.** Supporting Information

**Table S3.** Supporting Information

**Table S4.** Supporting Information

**Table S5.** Supporting Information

**Table S6.** Supporting Information

**Figure S1.** Supporting Information

**Figure S2.** Supporting Information

**Figure S3.** Supporting Information

**Figure S4.** Supporting Information

**Figure S5.** Supporting Information

**Figure S6.** Supporting Information

**Figure S7.** Supporting Information

**Figure S8.** Supporting Information

**Figure S9.** Supporting Information

**Figure S10.** Supporting Information

**Figure S11.** Supporting Information

**Figure S12.** Supporting Information

**Figure S13.** Supporting Information
