# Supplementary material for: Myogenesis modelled by human pluripotent stem cells: a multi‐omic study of Duchenne myopathy early onset
Source: J Cachexia Sarcopenia Muscle. 2021 Feb 14;12(1):209–32. doi: 10.1002/jcsm.12665 (PMC7890274; doi:10.1002/jcsm.12665)
Supplement: Supplementary file 1 — Data S1. Supporting Information [file JCSM-12-209-s001.pdf]

Supplementary references

DMD is initiated prior to the expression of skeletal muscle markers

PAX3 and *PAX7* [1]
GATA4 [2]
PAX8 [3]
TBX6 [4]
MIXL1 [5]
SOX17 [6]
SOX2 [7]
MEIS2 [8]
ACTA1 [9]
PAX3 [10]
NR2F2 [11],
PTN [12],
MET [13]
H19 and *IGF2* [14]
GLI3 [15]
GAS1 [16]
ZIC3 [17]
MET [18]
EPHA4 [19]
LBX1 [20]
FGF8 [21]
FOXD3 [22]
PAX1 [23]
EGFL6 [24]
SEMA6A [25]

DMD skeletal muscle progenitor cells exhibit specific muscle gene dysregulations

MYOD1 [26]
CDH15 [27]
TNNC2 [28]
SGCA [29]
RYR1 [30]
MIR1-1, MIR206 and MIR133 [31]
NGFR [32]
PAX7 and *CD34* [33]
MKX [34]
SOX5 [35]
SPP1 [36]
SALL1 [37]
OLIG2 [38]
MYOD1 [26]
ACTC1 [39]
ATP2A2 [40]
SNTA1 [41]

hiPSC differentiation leads to embryonic/foetal myotubes that reproduce DMD phenotypes

MIR1-1, *MIR133* and *MIR206* [42], [43]
MYOD1 [26]
MYOG [44]
CDH15 [27]
ITGA7 [45]
SGCA [29]
DTNA [46]
TNNC2 [28]
TNNT3 [47]
UNC45B [48]

NACA [49]
MUSK [50]
DOK7 [51]
MYH3 [52]
MYH8 [53]
MYL4 [54]
MYL5 [55]
MYH1 and *MYH2* [56]
SGCA [29]
SGCG [57]
RYR1 [30]
CACNA1S / CAV1.1 [58]
MEF2C [59]
TNNT3 [47]
RAPSN [60]
CKM [61]
MIR208a [62]
MYL7 [63]
RYR2 [64]
MYH11 [65]
CNN1 [66]
CHRNA3/B2/B4 [67]
MIR1-1, MIR206 and MIR133 [31]
SGCA [29]
SGCG [57]
UNC45B [48]
MYO18B [68]
CHRNB1 [69]
ATP2A2 [40]
RYR1 [30]

Markers of fibrosis are intrinsic to DMD hiPSC-derived myotubes

RRBP1 [70]
PLOD1 and *PLOD2* [71], [72]
SETD7 [73]
ANGPT1 [74]
CTGF [75]
COL1A2 [76]
MMPs and *TIMPs* [77]

[1] M. Buckingham and F. Relaix, “The role of Pax genes in the development of tissues and organs: Pax3 and Pax7 regulate muscle progenitor cell functions,” *Annu. Rev. Cell Dev. Biol.*, vol. 23, pp. 645–673, 2007.

[2] M. Heikinheimo, J. M. Scandrett, and D. B. Wilson, “Localization of Transcription Factor GATA-4 to Regions of the Mouse Embryo Involved in Cardiac Development,” *Dev. Biol.*, vol. 164, no. 2, pp. 361–373, 1994.

[3] P. L. Pfeffer, T. Gerster, K. Lun, M. Brand, and M. Busslinger, “Characterization of three novel members of the zebrafish Pax2/5/8 family: Dependency of Pax5 and Pax8 expression on the Pax2.1 (noi) function,” *Development*, vol. 125, no. 16, pp. 3063–3074, 1998.

[4] D. L. Chapman, A. Cooper-Morgan, Z. Harrelson, and V. E. Papaioannou, “Critical role for Tbx6 in mesoderm specification in the mouse embryo,” *Mech. Dev.*, vol. 120, no. 7, pp. 837–847, 2003.

[5] A. H. Hart *et al.*, “Mixl1 is required for axial mesendoderm morphogenesis and patterning in the murine embryo,” *Development*, vol. 129, no. 15, pp. 3597–3608, 2002.

[6] M. Kanai-Azuma *et al.*, “Depletion of definitive gut endoderm in Sox17-null mutant mice,” *Development*, vol. 129, no. 10, pp. 2367–2379, 2002.

[7] M. Rex *et al.*, “Dynamic expression of chicken Sox2 and Sox3 genes in ectoderm induced to form neural tissue,” *Dev. Dyn.*, vol. 209, no. 3, pp. 323–332, 1997.

[8] O. Machon, J. Masek, O. Machonova, S. Krauss, and Z. Kozmik, “Meis2 is essential for cranial and cardiac neural crest development,” *BMC Dev. Biol.*, vol. 15, no. 1, p. 40, 2015.

[9] N. G. Laing *et al.*, “Mutations and polymorphisms of the skeletal muscle α-actin gene (ACTA1),” *Hum. Mutat.*, vol. 30, no. 9, pp. 1267–1277, 2009.

[10] G. Kardon, T. A. Heanue, and C. J. Tabin, “Pax3 and Dach2 positive regulation in the developing somite,” *Dev. Dyn.*, vol. 224, no. 3, pp. 350–355, 2002.

[11] F. A. Pereira, Q. Yuhong, G. Zhou, M. J. Tsai, and S. Y. Tsai, “The orphan nuclear receptor COUP-TFII is required for angiogenesis and heart development,” *Genes Dev.*, vol. 13, no. 8, pp. 1037–1049, 1999.

[12] T. A. Mitsiadis *et al.*, “Expression of the heparin-binding cytokines, midkine (MK) and HB-GAM (pleiotrophin) is associated with epithelial-mesenchymal interactions during fetal development and organogenesis,” *Development*, vol. 121, no. 1, pp. 37–51, 1995.

[13] X. M. Yang, K. Vogan, P. Gros, and M. Park, “Expression of the met receptor tyrosine kinase in muscle progenitor cells in somites and limbs is absent in Splotch mice,” *Development*, vol. 122, no. 7, pp. 2163–2171, 1996.

[14] H. Sasaki, A. C. Ferguson-Smith, A. S. W. Shum, S. C. Barton, and M. A. Surani, “Temporal and spatial regulation of H19 imprinting in normal and uniparental mouse embryos,” *Development*, vol. 121, no. 12, pp. 4195–4202, 1995.

[15] A. G. Borycki, L. Mendham, and C. P. Emerson, “Control of somite patterning by Sonic hedgehog and its downstream signal response genes,” *Development*, vol. 125, no. 4, pp. 777–790, 1998.

[16] C. S. Lee, L. Buttitta, and C. M. Fan, “Evidence that the WNT-inducible growth arrest-specific gene 1 encodes an antagonist of sonic hedgehog signaling in the somite,” *Proc. Natl. Acad. Sci. U. S. A.*, vol. 98, no. 20, pp. 11347–11352, 2001.

[17] A. R. McMahon and C. S. Merzdorf, “Expression of the zic1, zic2, zic3, and zic4 genes in early chick embryos,” *BMC Res. Notes*, vol. 3, no. 1, p. 167, 2010.

[18] F. Bladt, D. Riethmacher, S. Isenmann, A. Aguzzi, and C. Birchmeier, “Essential role for the c-met receptor in the migration of myogenic precursor cells into the limb bud,” *Nature*, vol. 376, no. 6543, pp. 768–771, 1995.

[19] M. E. Swartz, J. Eberhart, E. B. Pasquale, and C. E. Krull, “EphA4/ephrin-A5 interactions in muscle precursor cell migration in the avian forelimb,” *Development*, vol. 128, no. 23, pp. 4669–4680, 2001.

[20] K. Schäfer and T. Braun, “Early specification of limb muscle precursor cells by the homeobox gene Lbx1h,” *Nat. Genet.*, vol. 23, no. 2, pp. 213–216, 1999.

[21] P. H. Crossley, G. Minowada, C. A. MacArthur, and G. R. Martin, “Roles for FGF8 in the induction, initiation, and maintenance of chick limb development,” *Cell*, vol. 84, no. 1, pp. 127–136, 1996.

[22] R. A. Stewart *et al.*, “Zebrafish foxd3 is selectively required for neural crest specification, migration and survival,” *Dev. Biol.*, vol. 292, no. 1, pp. 174–188, 2006.

[23] U. Deutsch, G. R. Dressler, and P. Gruss, “Pax 1, a member of a paired box homologous murine gene family, is expressed in segmented structures during development,” *Cell*, vol. 53, no. 4, pp. 617–625, 1988.

[24] G. Buchner *et al.*, “MAEG, an EGF-repeat containing gene, is a new marker associated with dermatome specification and morphogenesis of its derivatives,” *Mech. Dev.*, vol. 98, no. 1–2, pp. 179–182, 2000.

[25] X. M. Xu *et al.*, “The transmembrane protein semaphorin 6A repels embryonic sympathetic axons,” *J. Neurosci.*, vol. 20, no. 7, pp. 2638–2648, 2000.

[26] R. L. Davis, H. Weintraub, and A. B. Lassar, “Expression of a single transfected cDNA converts fibroblasts to myoblasts,” *Cell*, vol. 51, no. 6, pp. 987–1000, 1987.

[27] M. Donalies, M. Cramer, M. Ringwald, and A. Starzinski-Powitz, “Expression of M-cadherin, a member of the cadherin multigene family, correlates with differentiation of skeletal muscle cells,” *Proc. Natl. Acad. Sci. U. S. A.*, vol. 88, no. 18, pp. 8024–8028, 1991.

[28] R. Gahlmann and L. Kedes, “Cloning, structural analysis, and expression of the human fast twitch skeletal muscle troponin C gene,” *J. Biol. Chem.*, vol. 265, no. 21, pp. 12520–12528, 1990.

[29] S. L. Roberds, R. D. Anderson, O. Ibraghimov-Beskrovnaya, and K. P. Campbell, “Primary structure and muscle-specific expression of the 50-kDa dystrophin- associated glycoprotein (adhalin),” *J. Biol. Chem.*, vol. 268, no. 32, pp. 23739–23742, 1993.

[30] A. E. MacKenzie *et al.*, “The human ryanodine receptor gene: Its mapping to 19q13.1, placement in a chromosome 19 linkage group, and exclusion as the gene causing myotonic dystrophy,” *Am. J. Hum. Genet.*, vol. 46, no. 6, pp. 1082–1089, 1990.

[31] S. Greco *et al.*, “Common micro-RNA signature in skeletal muscle damage and regeneration induced by Duchenne muscular dystrophy and acute ischemia,” *FASEB J.*, vol. 23, no. 10, pp. 3335–3346, 2009.

[32] M. R. Hicks *et al.*, “ERBB3 and NGFR mark a distinct skeletal muscle progenitor cell in human development and hPSCs,” *Nat. Cell Biol.*, vol. 20, no. 1, pp. 46–57, 2018.

[33] D. Montarras *et al.*, “Developmental biology: Direct isolation of satellite cells for skeletal muscle regeneration,” *Science (80-.).*, vol. 309, no. 5743, pp. 2064–2067, 2005.

[34] C. Milet and D. Duprez, “The Mkx homeoprotein promotes tenogenesis in stem cells and improves tendon repair,” *Ann. Transl. Med.*, vol. 3, no. Suppl 1, p. S33, 2015.

[35] V. Lefebvre, P. Li, and B. De Crombrugghe, “A new long form of Sox5 (L-Sox5), Sox6 and Sox9 are coexpressed in chondrogenesis and cooperatively activate the type II collagen gene,” *EMBO J.*, vol. 17, no. 19, pp. 5718–5733, 1998.

[36] M. NODA and D. T. DENHARDT, “Regulation of Osteopontin Gene Expression in Osteoblasts,” *Ann. N. Y. Acad. Sci.*, vol. 760, no. 1, pp. 242–248, 1995.

[37] A. Atala, “Re: Sall1 Maintains Nephron Progenitors and Nascent Nephrons by Acting as Both an Activator and a Repressor: Editorial Comment,” *J. Urol.*, vol. 194, no. 2, pp. 592–593, 2015.

[38] C. Santiago and J. Bashaw, “Transcription factors and effectors that regulate neuronal morphology,” *Dev.*, vol. 141, no. 24, pp. 4667–4680, 2014.

[39] J. K. Boutilier *et al.*, “Variable cardiac α-actin (Actc1) expression in early adult skeletal muscle correlates with promoter methylation,” *Biochim. Biophys. Acta - Gene Regul. Mech.*, vol. 1860, no. 10, pp. 1025–1036, 2017.

[40] J. Lytton and D. H. MacLennan, “Molecular cloning of cDNAs from human kidney coding for two alternatively spliced products of the cardiac Ca2+-ATPase gene,” *J. Biol. Chem.*, vol. 263, no. 29, pp. 15024–15031, 1988.

[41] M. Yoshida and E. Ozawa, “Glycoprotein complex anchoring dystrophin to sarcolemma,” *J. Biochem.*, vol. 108, no. 5, pp. 748–752, 1990.

[42] J. F. Chen *et al.*, “The role of microRNA-1 and microRNA-133 in skeletal muscle proliferation and differentiation,” *Nat. Genet.*, vol. 38, no. 2, pp. 228–233, 2006.

[43] K. K. Hak, S. L. Yong, U. Sivaprasad, A. Malhotra, and A. Dutta, “Muscle-specific microRNA miR-206 promotes muscle differentiation,” *J. Cell Biol.*, vol.

174, no. 5, pp. 677–687, 2006.
[44] P. Hasty *et al.*, “Muscle deficiency and neonatal death in mice with a targeted mutation in the myogenin gene,” *Nature*, vol. 364, no. 6437, pp. 501–506, 1993.
[45] N. Vignier, B. Moghadaszadeh, F. Gary, J. Beckmann, U. Mayer, and P. Guicheney, “Structure, genetic localization, and identification of the cardiac and skeletal muscle transcripts of the human integrin α7 gene (ITGA7),” *Biochem. Biophys. Res. Commun.*, vol. 260, no. 2, pp. 357–364, 1999.
[46] S. E. Newey *et al.*, “Syncoilin, a Novel Member of the Intermediate Filament Superfamily That Interacts with α-Dystrobrevin in Skeletal Muscle,” *J. Biol. Chem.*, vol. 276, no. 9, pp. 6645–6655, 2001.
[47] qi L. wu, P. K. Jha, M. K. Raychowdhury, Y. du, P. C. Leavis, and S. Sarkar, “Isolation and Characterization of Human Fast Skeletal β Troponin T cDNA: Comparative Sequence Analysis of Isoforms and Insight into the Evolution of Members of a Multigene Family,” *DNA Cell Biol.*, vol. 13, no. 3, pp. 217–233, 1994.
[48] E. P. Bernick, P. J. Zhang, and S. Du, “Knockdown and overexpression of Unc-45b result in defective myofibril organization in skeletal muscles of zebrafish embryos,” *BMC Cell Biol.*, vol. 11, no. 1, p. 70, 2010.
[49] H. Li, W. R. Randall, and S.-J. Du, “skNAC (skeletal Naca), a muscle-specific isoform of Naca (nascent polypeptide-associated complex alpha), is required for myofibril organization,” *FASEB J.*, vol. 23, no. 6, pp. 1988–2000, 2009.
[50] T. M. DeChiara *et al.*, “The receptor tyrosine kinase MuSK is required for neuromuscular junction formation in vivo,” *Cell*, vol. 85, no. 4, pp. 501–512, 1996.
[51] K. Okada *et al.*, “The muscle protein Dok-7 is essential for neuromuscular synaptogenesis,” *Science (80-.).*, vol. 312, no. 5781, pp. 1802–1805, 2006.
[52] I. Karsch-Mizrachi, M. Travis, H. Blau, and L. A. Leinwand, “Expression and DNA sequence analysis of a human embryonic skeletal muscle mvosin heavy chain gene,” *Nucleic Acids Res.*, vol. 17, no. 15, pp. 6167–6179, 1989.
[53] A. Weiss, S. Schiaffino, and L. A. Leinwand, “Comparative sequence analysis of the complete human sarcomeric myosin heavy chain family: Implications for functional diversity,” *J. Mol. Biol.*, vol. 290, no. 1, pp. 61–75, 1999.
[54] R. C. Strohman, J. Micou-Eastwood, C. A. Glass, and R. Matsuda, “Human fetal muscle and cultured myotubes derived from it contain a fetal-specific myosin light chain,” *Science (80-.).*, vol. 221, no. 4614, pp. 955–957, 1983.
[55] C. Collins, K. Schappert, and M. R. Hayden, “The genomic organization of a novel regulatory myosin light chain gene (MYL5) that maps to chromosome 4p16.3 and shows different patterns of expression between primates,” *Hum. Mol. Genet.*, vol. 1, no. 9, pp. 727–733, 1992.
[56] V. Smerdu, I. Karsch-Mizrachi, M. Campione, L. Leinwand, and S. Schiaffino, “Type IIx myosin heavy chain transcripts are expressed in type IIb fibers of human skeletal muscle,” *Am. J. Physiol. - Cell Physiol.*, vol. 267, no. 6 36-6, pp. C1723-8, 1994.
[57] S. Noguchi *et al.*, “Mutations in the dystrophin-associated protein γ-sarcoglycan in chromosome 13 muscular dystrophy,” *Science (80-.).*, vol. 270, no. 5237, pp. 819–822, 1995.
[58] K. P. Campbell, A. T. Leung, and A. H. Sharp, “The biochemistry and molecular biology of the dihydropyridine-sensitive calcium channel,” *Trends Neurosci.*, vol. 11, no. 10, pp. 425–430, 1988.
[59] R. E. Breitbart, C. Liang -s., L. B. Smoot, D. A. Laheru, V. Mahdavi, and B. Nadal-Ginard, “A fourth human MEF2 transcription factor, hMEF2D, is an early marker of the myogenic lineage,” *Development*, vol. 118, no. 4, pp. 1095–1106, 1993.
[60] M. Gautam *et al.*, “Failure of postsynaptic specialization to develop at neuromuscular junctions of rapsyn-deficient mice,” *Nature*, vol. 377, no. 6546, pp. 232–236, 1995.
[61] D. M. Dawson, H. M. Eppenberger, and M. E. Eppenberger, “Multiple Molecular Forms of Creatine Kinases,” *Ann. N. Y. Acad. Sci.*, vol. 151, no. 1, pp. 616–626, 1968.
[62] E. Van Rooij, L. B. Sutherland, X. Qi, J. A. Richardson, J. Hill, and E. N. Olson, “Control of stress-dependent cardiac growth and gene expression by a microRNA,” *Science (80-.).*, vol. 316, no. 5824, pp. 575–579, 2007.
[63] D. Hailstones *et al.*, “Differential regulation of the atrial isoforms of the myosin light chains during striated muscle development,” *J. Biol. Chem.*, vol. 267, no. 32, pp. 23295–23300, 1992.
[64] S. O. Marx *et al.*, “PKA phosphorylation dissociates FKBP12.6 from the calcium release channel (ryanodine receptor): Defective regulation in failing hearts,” *Cell*, vol. 101, no. 4, pp. 365–376, 2000.
[65] M. Kuro-o *et al.*, “Developmentally regulated expression of vascular smooth muscle myosin heavy chain isoforms,” *J. Biol. Chem.*, vol. 264, no. 31, pp. 18272–18275, 1989.
[66] M. Gimona, M. Herzog, J. Vandekerckhove, and J. V. Small, “Smooth muscle specific expression of calponin,” *FEBS Lett.*, vol. 274, no. 1–2, pp. 159–162, 1990.
[67] R. M. Eglen, H. Reddy, N. Watson, and R. A. J. Challiss, “Muscarinic acetylcholine receptor subtypes in smooth muscle,” *Trends Pharmacol. Sci.*, vol. 15, no. 4, pp. 114–119, 1994.
[68] R. Ajima *et al.*, “Deficiency of Myo18B in mice results in embryonic lethality with cardiac myofibrillar aberrations,” *Genes to Cells*, vol. 13, no. 10, pp. 987–999, 2008.
[69] O. Heidmann *et al.*, “Chromosomal localization of muscle nicotinic acetylcholine receptor genes in the mouse,” *Science (80-.).*, vol. 234, no. 4778, pp. 866–868, 1986.
[70] T. Ueno *et al.*, “Enhancement of procollagen biosynthesis by p180 through augmented ribosome association on the endoplasmic reticulum in response to stimulated secretion,” *J. Biol. Chem.*, vol. 285, no. 39, pp. 29941–29950, 2010.
[71] T. Hautala, M. G. Byers, R. L. Eddy, T. B. Shows, K. I. Kivirikko, and R. Myllyla, “Cloning of human lysyl hydroxylase: Complete cDNA-derived amino acid sequence and assignment of the gene (PLOD) to chromosome 1p36.3→p36.2,” *Genomics*, vol. 13, no. 1, pp. 62–69, 1992.
[72] M. Valtavaara, H. Papponen, A. M. Pirttilä, K. Hiltunen, H. Helander, and R. Myllylä, “Cloning and characterization of a novel human lysyl hydroxylase isoform highly expressed in pancreas and muscle,” *J. Biol. Chem.*, vol. 272, no. 11, pp. 6831–6834, 1997.
[73] J. H. A. Martens, M. Verlaan, E. Kalkhoven, and A. Zantema, “Cascade of Distinct Histone Modifications during Collagenase Gene Activation,” *Mol. Cell. Biol.*, vol. 23, no. 5, pp. 1808–1816, 2003.
[74] D. A. Long *et al.*, “Angiopoietin-1 therapy enhances fibrosis and inflammation following folic acid-induced acute renal injury,” *Kidney Int.*, vol. 74, no. 3, pp. 300–309, 2008.
[75] K. E. Lipson, C. Wong, Y. Teng, and S. Spong, “CTGF is a central mediator of tissue remodeling and fibrosis and its inhibition can reverse the process of fibrosis,” *Fibrogenesis Tissue Repair*, vol. 5, no. S1, p. S24, 2012.
[76] M. Fragiadaki *et al.*, “Interstitial fibrosis is associated with increased COL1A2 transcription in AA-injured renal tubular epithelial cells in vivo,” *Matrix Biol.*, vol. 30, no. 7–8, pp. 396–403, 2011.
[77] S. Hemmann, J. Graf, M. Roderfeld, and E. Roeb, “Expression of MMPs and TIMPs in liver fibrosis - a systematic review with special emphasis on anti-fibrotic strategies,” *J. Hepatol.*, vol. 46, no. 5, pp. 955–975, 2007.
