# Supplementary material for: Myogenesis modelled by human pluripotent stem cells: a multi‐omic study of Duchenne myopathy early onset
Source: J Cachexia Sarcopenia Muscle. 2021 Feb 14;12(1):209–32. doi: 10.1002/jcsm.12665 (PMC7890274; doi:10.1002/jcsm.12665)
Supplement: Supplementary file 8 — Figure S1. Supporting Information [file JCSM-12-209-s008.pdf]

Figure S1

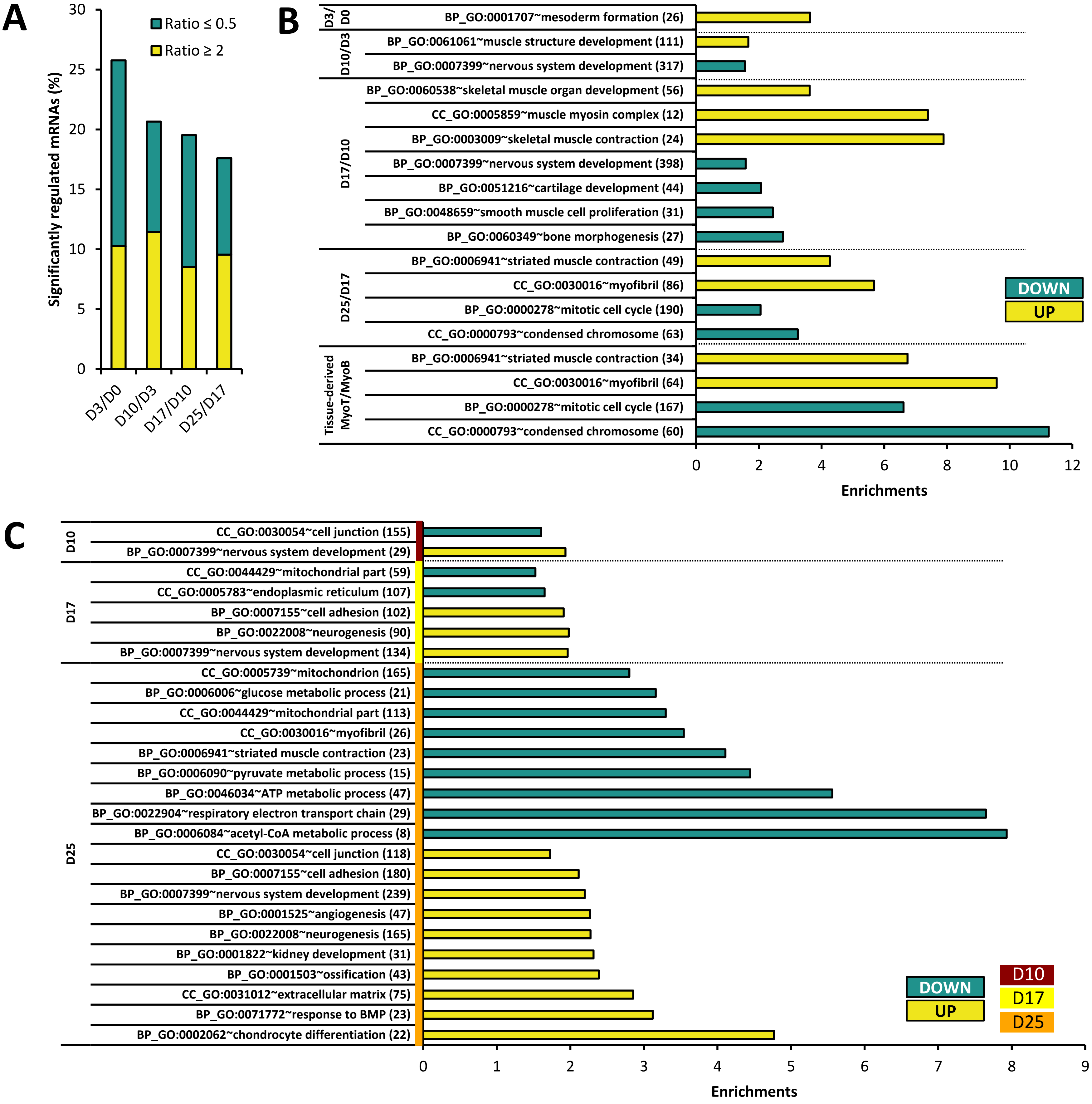

Figure S1 – Gene ontology enrichments over the course of healthy and DMD hiPSC differentiation A) Proportions of significantly regulated mRNAs (adjusted p-value ≤ 0.01) between successive differentiation time points during the differentiation of healthy hiPSCs. Gene ontology enrichments on **B)** significantly regulated terms between successive differentiation time points in healthy cells (number of genes in brackets) and C) significantly dysregulated terms at each differentiation time points in DMD cells. The number of genes involved in these significant enrichments is indicated in brackets next to each GO term. In green, GO terms related to downregulated genes and in yellow, GO terms related to upregulated genes (BP: biological process; CC: cellular component; D: day; hiPSC: human induced pluripotent stem cell; MyoB: myoblast; MyoT: myotube).
