# Supplementary material for: Myogenesis modelled by human pluripotent stem cells: a multi‐omic study of Duchenne myopathy early onset
Source: J Cachexia Sarcopenia Muscle. 2021 Feb 14;12(1):209–32. doi: 10.1002/jcsm.12665 (PMC7890274; doi:10.1002/jcsm.12665)
Supplement: Supplementary file 9 — Figure S2. Supporting Information [file JCSM-12-209-s009.pdf]

Figure S2

A

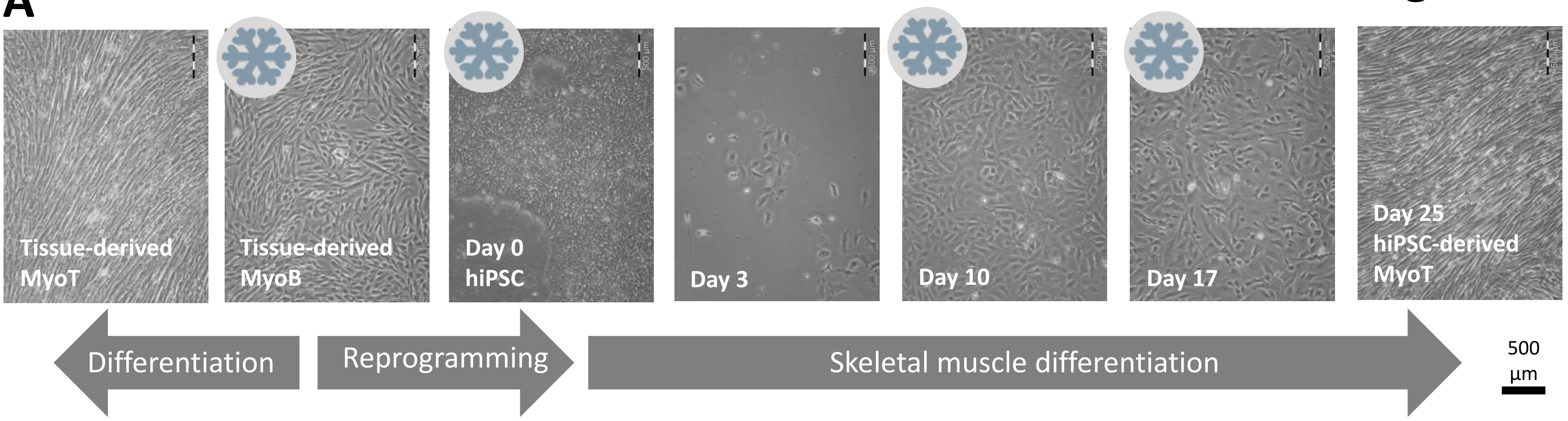

B

Target Name	Cell state	Phenotype	Ct mean	Ct SEM
UBC (reference gene)	D0	Healthy	23.7	0.1
		DMD	24.5	0.3
	D3	Healthy	24.9	0.3
		DMD	24.8	0.3
	D10	Healthy	24.9	0.2
		DMD	25.5	0.4
	D17	Healthy	25.7	0.6
		DMD	26.0	0.7
	D25	Healthy	25.8	0.4
		DMD	27.1	1.4
Dp71-40	D0	Healthy	25.2	0.3
		DMD	26.1	0.0
	D3	Healthy	25.2	0.2
		DMD	26.4	0.3
	D10	Healthy	24.5	0.7
		DMD	24.5	0.2
	D17	Healthy	27.2	0.5
		DMD	26.5	0.4
	D25	Healthy	26.6	0.8
		DMD	25.9	1.9
Dp116	D3	Healthy	35.9	0.3
	D10	Healthy	33.6	2.3
		DMD	35.0	0.5
	D17	Healthy	37.9	2.6
		DMD	35.6	0.4
	D25	Healthy	34.2	1.2
		DMD	35.3	2.5
Dp140	D0	Healthy	32.1	1.6
		DMD	33.8	0.7
	D3	Healthy	32.7	0.7
		DMD	36.5	0.2
	D10	Healthy	32.7	2.8
		DMD	34.4	0.7
	D25	Healthy	36.5	0.2
Dp260	D0	Healthy	31.2	0.5
		DMD	31.6	1.0
	D3	Healthy	29.3	1.4
		DMD	30.0	2.3
	D10	Healthy	30.0	1.0
		DMD	30.3	0.6
	D17	Healthy	25.3	1.2
		DMD	26.4	0.3
	D25	Healthy	22.8	0.4
		DMD	25.1	2.5
Dp412e	D0	Healthy	37.2	1.7
		DMD	35.8	2.4
	D3	Healthy	33.3	1.9
		DMD	32.7	2.1
Dp427c	D0	Healthy	31.6	0.8
		DMD	33.3	0.8
	D3	Healthy	34.1	0.2
		DMD	38.2	3.8
	D10	Healthy	33.5	0.9
		DMD	34.8	0.4
	D17	Healthy	30.1	1.0
		DMD	30.8	0.4
	D25	Healthy	30.1	0.3
		DMD	30.0	0.3
Dp427m	D3	Healthy	35.2	0.9
		DMD	34.7	0.9
	D10	Healthy	28.5	1.2
		DMD	29.4	0.8
	D17	Healthy	22.5	1.1
		DMD	23.6	0.1
	D25	Healthy	20.4	0.4
		DMD	21.9	1.4

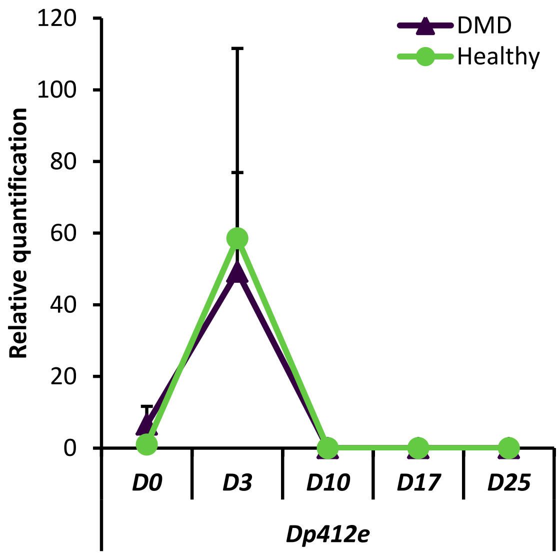

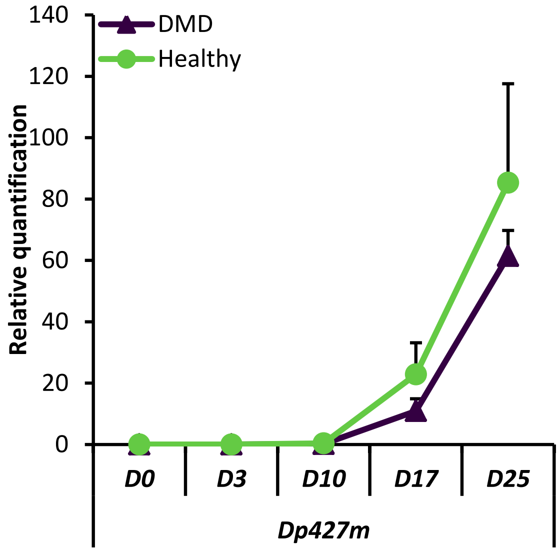

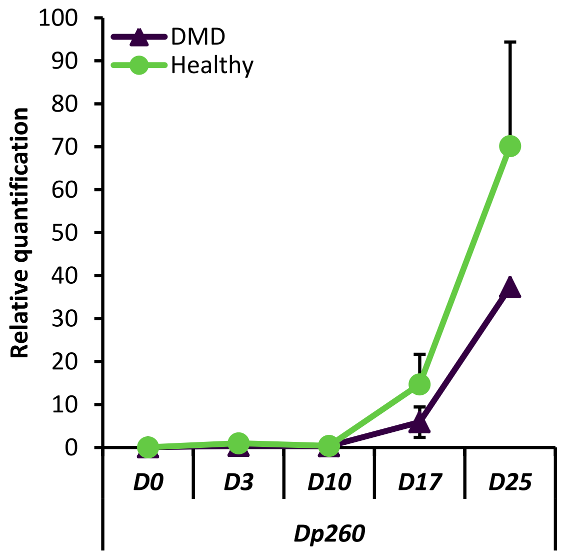

Figure S2 – DMD variant expression over the course of hiPSC differentiation. A) Bright field microscope pictures at the 7 differentiation points giving rise to hiPSC-derived and tissue-derived MyoT. Possible cryopreservation time points are indicated by snowflakes. **B)** RT-qPCR relative quantification of *DMD* variants expression during differentiation of hiPSCs (D0) into MyoT (D25) with the related cycle threshold (CT) values (Ct: cycle threshold; D: day; hiPSC: human induced pluripotent stem cell; MyoB: myoblast; MyoT: myotube).
