# Supplementary material for: Myogenesis modelled by human pluripotent stem cells: a multi‐omic study of Duchenne myopathy early onset
Source: J Cachexia Sarcopenia Muscle. 2021 Feb 14;12(1):209–32. doi: 10.1002/jcsm.12665 (PMC7890274; doi:10.1002/jcsm.12665)
Supplement: Supplementary file 10 — Figure S3. Supporting Information [file JCSM-12-209-s010.pdf]

Figure S3

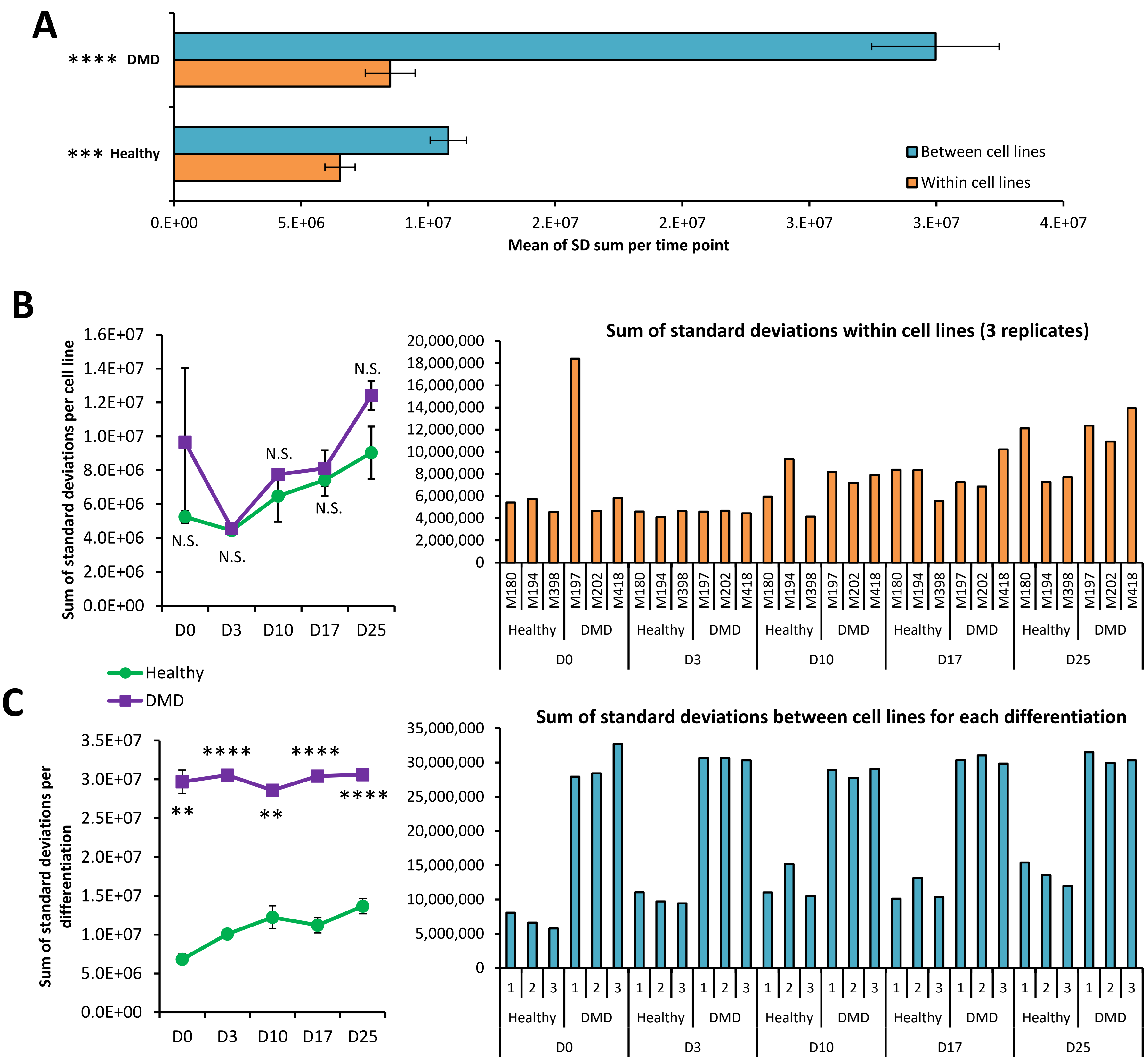

Figure S3 – Measures of gene expression standard deviations in hiPSC-derived culture. A) Mean of the sum per time point of all gene expression standard deviations in healthy and DMD cells, either considering triplicates per cell line (within cell lines, orange) or per differentiation (between cell lines, blue), two-tailed Mann-Whitney t-test. Sum per time point of gene expression standard deviations in healthy and DMD cells **B)** per cell line or **C)** per differentiation. **B)C)** Right panels give detailed data while left panels give aggregated data with two-tailed unpaired t-test (*p-value ≤ 0.05, **p-value ≤ 0.01, ***p-value ≤ 0.001, ****p-value ≤ 0.0001, N.S.: not significant; D: day).
