# Supplementary material for: Myogenesis modelled by human pluripotent stem cells: a multi‐omic study of Duchenne myopathy early onset
Source: J Cachexia Sarcopenia Muscle. 2021 Feb 14;12(1):209–32. doi: 10.1002/jcsm.12665 (PMC7890274; doi:10.1002/jcsm.12665)
Supplement: Supplementary file 11 — Figure S4. Supporting Information [file JCSM-12-209-s011.pdf]

Figure S4

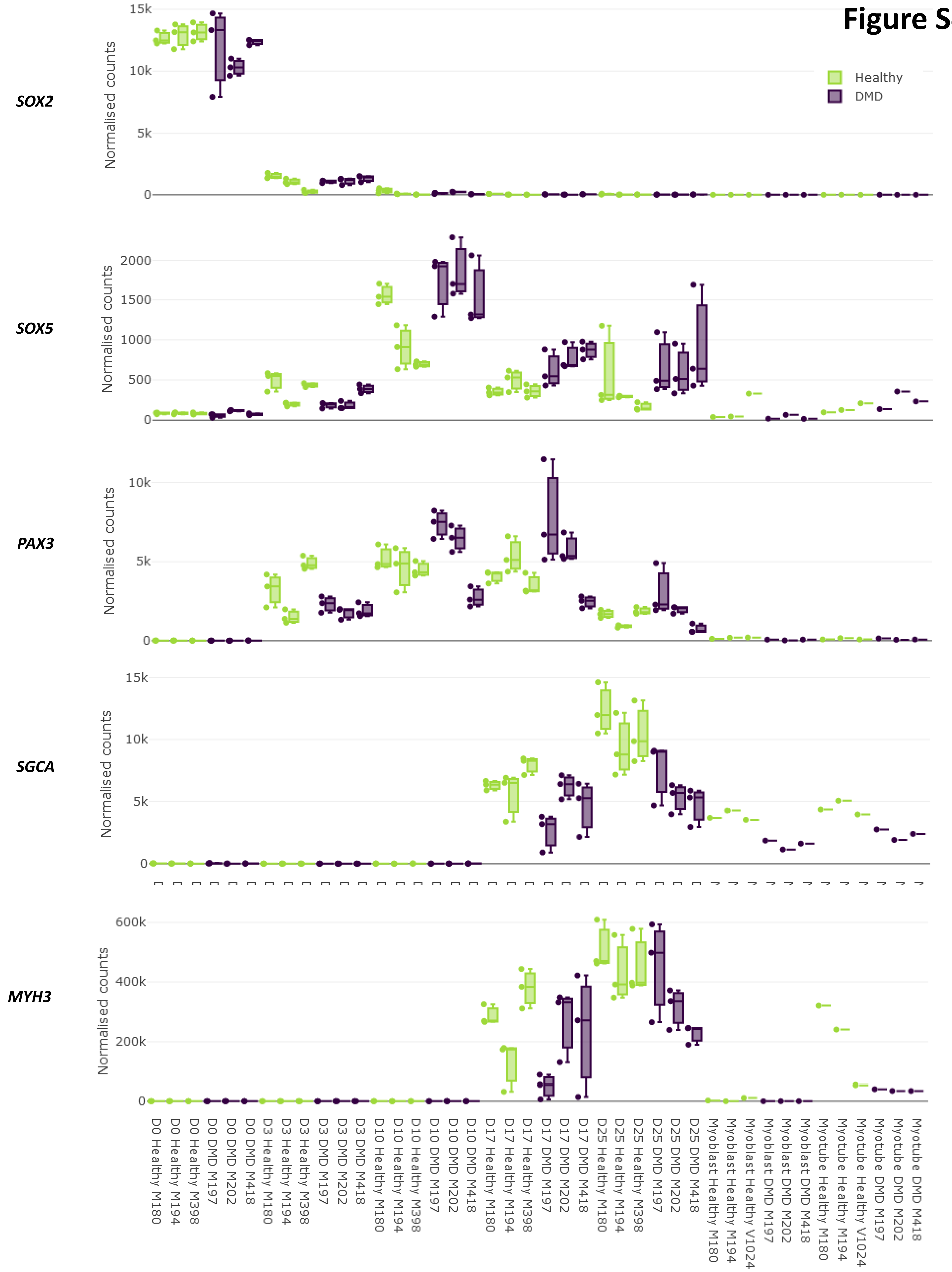

Figure S4 – Examples of detailed cell gene expression. Boxplots of RNA-seq expression data at each time point for *SOX2*, *SOX5*, *PAX3*, *SGCA* and *MYH3* (D: day).
