# Supplementary material for: Myogenesis modelled by human pluripotent stem cells: a multi‐omic study of Duchenne myopathy early onset
Source: J Cachexia Sarcopenia Muscle. 2021 Feb 14;12(1):209–32. doi: 10.1002/jcsm.12665 (PMC7890274; doi:10.1002/jcsm.12665)
Supplement: Supplementary file 12 — Figure S5. Supporting Information [file JCSM-12-209-s012.pdf]

Figure S5

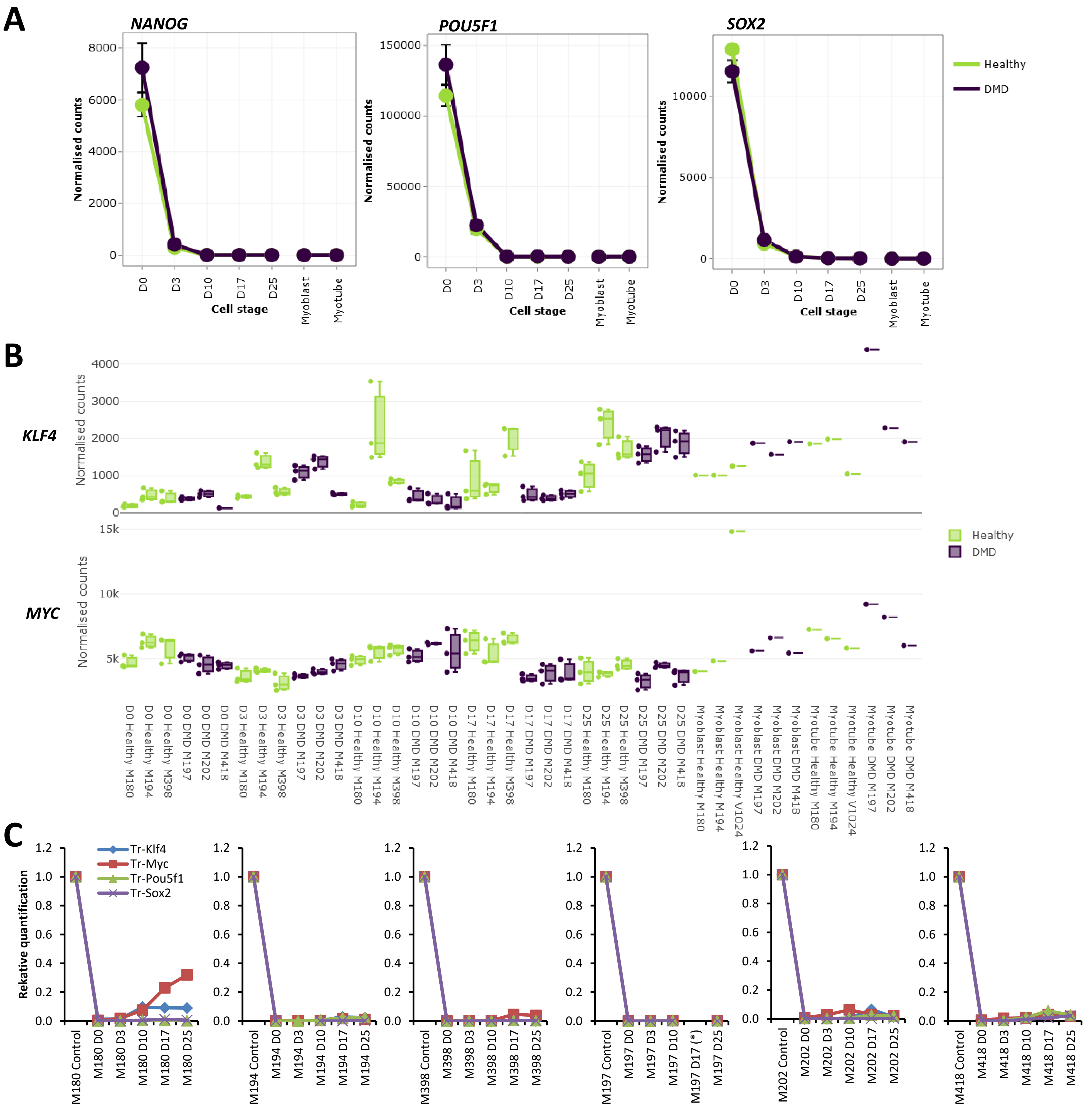

D

	D17			D25		
	1 vs 2	1 vs 3	2 vs 3	1 vs 2	1 vs 3	2 vs 3
Mean	0.96	0.97	0.96	0.97	0.96	0.97
SD	0.004	0.002	0.003	0.005	0.005	0.004

Figure S5 – Gene expression of both pluripotency and reprogramming genes during differentiation of hiPSCs and in adult tissue-derived cells. Gene expression from RNA-seq analysis of **A)** pluripotency markers *Nanog*, *Pou5f1* and *Sox2*, as well as **B)** reprogramming genes *Klf4* and *Myc*. **C)** qPCR quantification of transgenes *Klf4*, *Myc*, *Pou5f1* and *Sox2*. Positive controls are samples from day 4 post-reprogramming. **D)** Spearman correlations between healthy cell lines at day 17 and day 25.
