# Supplementary material for: Myogenesis modelled by human pluripotent stem cells: a multi‐omic study of Duchenne myopathy early onset
Source: J Cachexia Sarcopenia Muscle. 2021 Feb 14;12(1):209–32. doi: 10.1002/jcsm.12665 (PMC7890274; doi:10.1002/jcsm.12665)
Supplement: Supplementary file 13 — Figure S6. Supporting Information [file JCSM-12-209-s013.pdf]

Figure S6

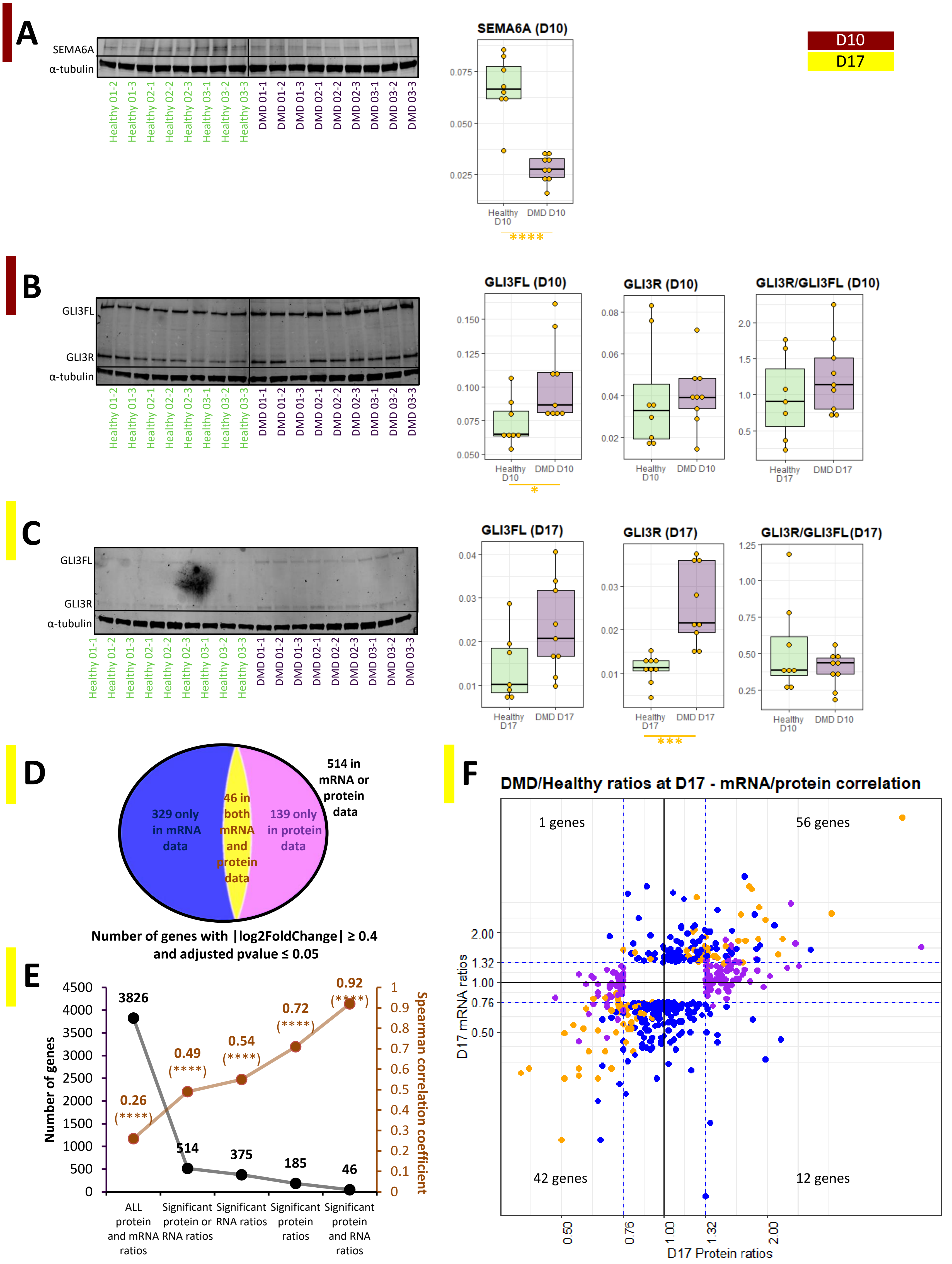

Figure S6 – Comparison of healthy and DMD cells at D10 and D17, protein analyses. Western blots and quantifications of **A)** SEMA6A at D10, **B)** GLI3 at D10 and **C)** GLI3 at D17. Omics comparison of mRNA and protein data at day 17: **D)** Venn diagram of the number of genes with |log2FoldChange| ≥ 0.4 and adjusted pvalue ≤ 0.05 in either transcriptomic or proteomic data, **E)** their associated Spearman correlation coefficient in brown, as well as **D)** their correlation graph with the number of genes with |log2FoldChange| ≥ 0.4 in both sets are indicated (genes with p-value ≥ 0.05 only in transcriptomics are in blue, only in proteomics in purple and in both in orange). (*p-value ≤ 0.05, **p-value ≤ 0.01, ***p-value ≤ 0.001, ****p-value ≤ 0.0001; D: day; GLI3FL: GLI3 full length; GLI3R: GLI3 repressor).
