# Supplementary material for: Myogenesis modelled by human pluripotent stem cells: a multi‐omic study of Duchenne myopathy early onset
Source: J Cachexia Sarcopenia Muscle. 2021 Feb 14;12(1):209–32. doi: 10.1002/jcsm.12665 (PMC7890274; doi:10.1002/jcsm.12665)
Supplement: Supplementary file 14 — Figure S7. Supporting Information [file JCSM-12-209-s014.pdf]

Figure S7

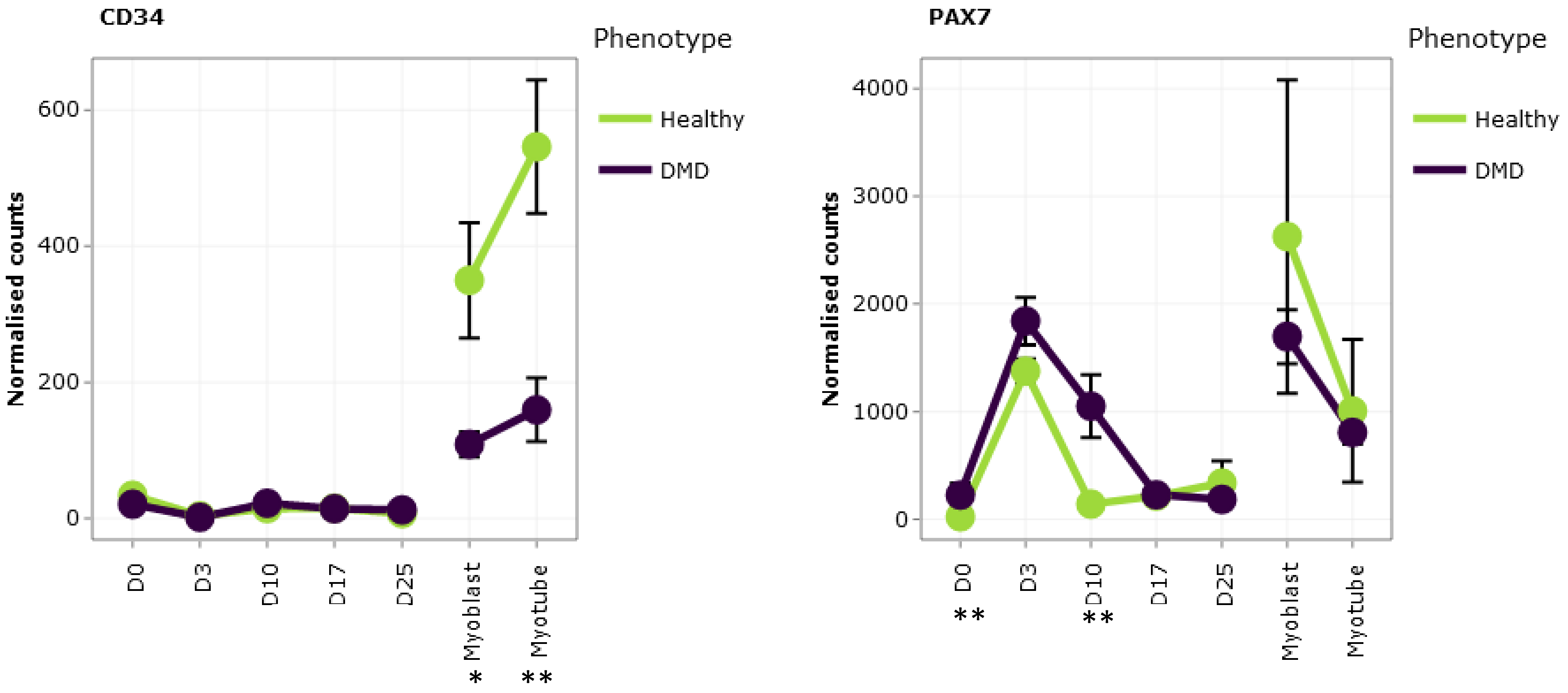

Figure S7 – Gene expression of skeletal stem cell muscle markers. RNA-seq expression data of *CD34* and *PAX7* (*adjusted p-value ≤ 0.05, **adjusted p-value ≤ 0.01, ***adjusted p-value ≤ 0.001, ****adjusted p-value ≤ 0.0001; D: day).
