# Supplementary material for: Myogenesis modelled by human pluripotent stem cells: a multi‐omic study of Duchenne myopathy early onset
Source: J Cachexia Sarcopenia Muscle. 2021 Feb 14;12(1):209–32. doi: 10.1002/jcsm.12665 (PMC7890274; doi:10.1002/jcsm.12665)
Supplement: Supplementary file 15 — Figure S8. Supporting Information [file JCSM-12-209-s015.pdf]

Figure S8

A

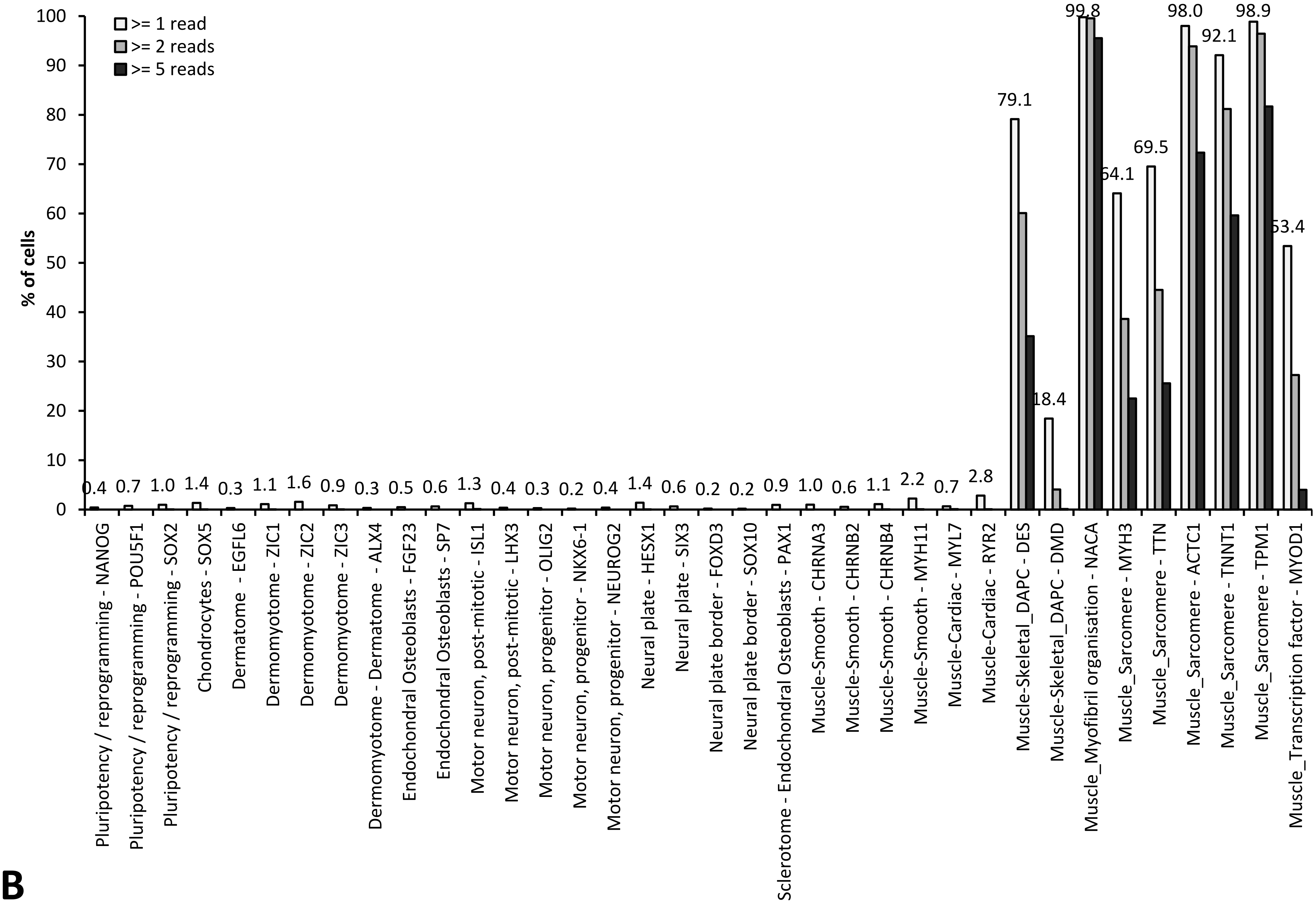

B

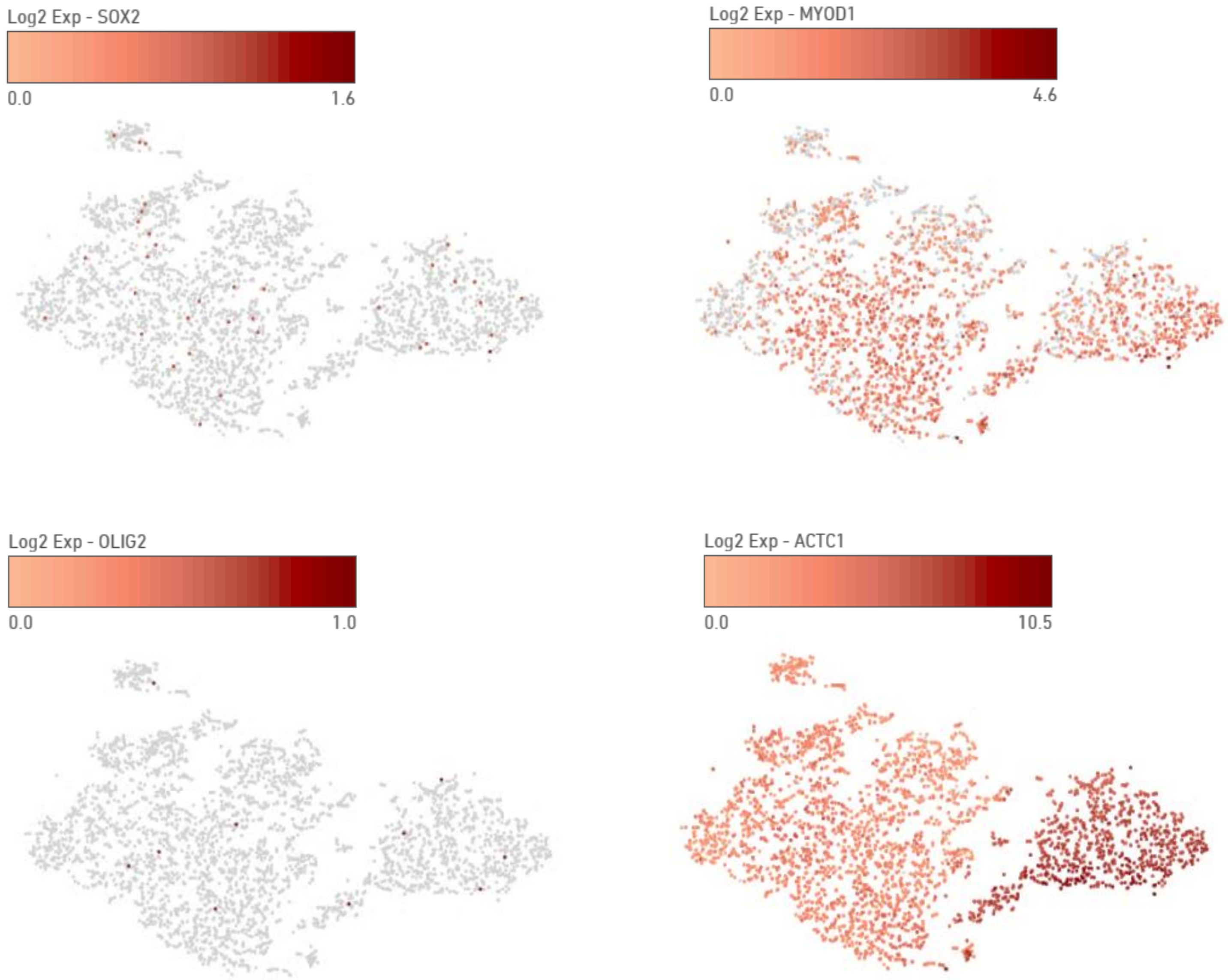

Figure S8 – Single-cell gene expression at day 17. The mean reads per cell was 62,171 and the median genes per cell was 4,040. **A)** Selection of genes with the percentage of cells having more than 1, 2 or 5 reads. **B)** t-SNE representing the $\log_2$ expression levels of *SOX2*, *OLIG2*, *MYOD1* and *ACTC1*.
