# Supplementary material for: Myogenesis modelled by human pluripotent stem cells: a multi‐omic study of Duchenne myopathy early onset
Source: J Cachexia Sarcopenia Muscle. 2021 Feb 14;12(1):209–32. doi: 10.1002/jcsm.12665 (PMC7890274; doi:10.1002/jcsm.12665)
Supplement: Supplementary file 16 — Figure S9. Supporting Information [file JCSM-12-209-s016.pdf]

Figure S9

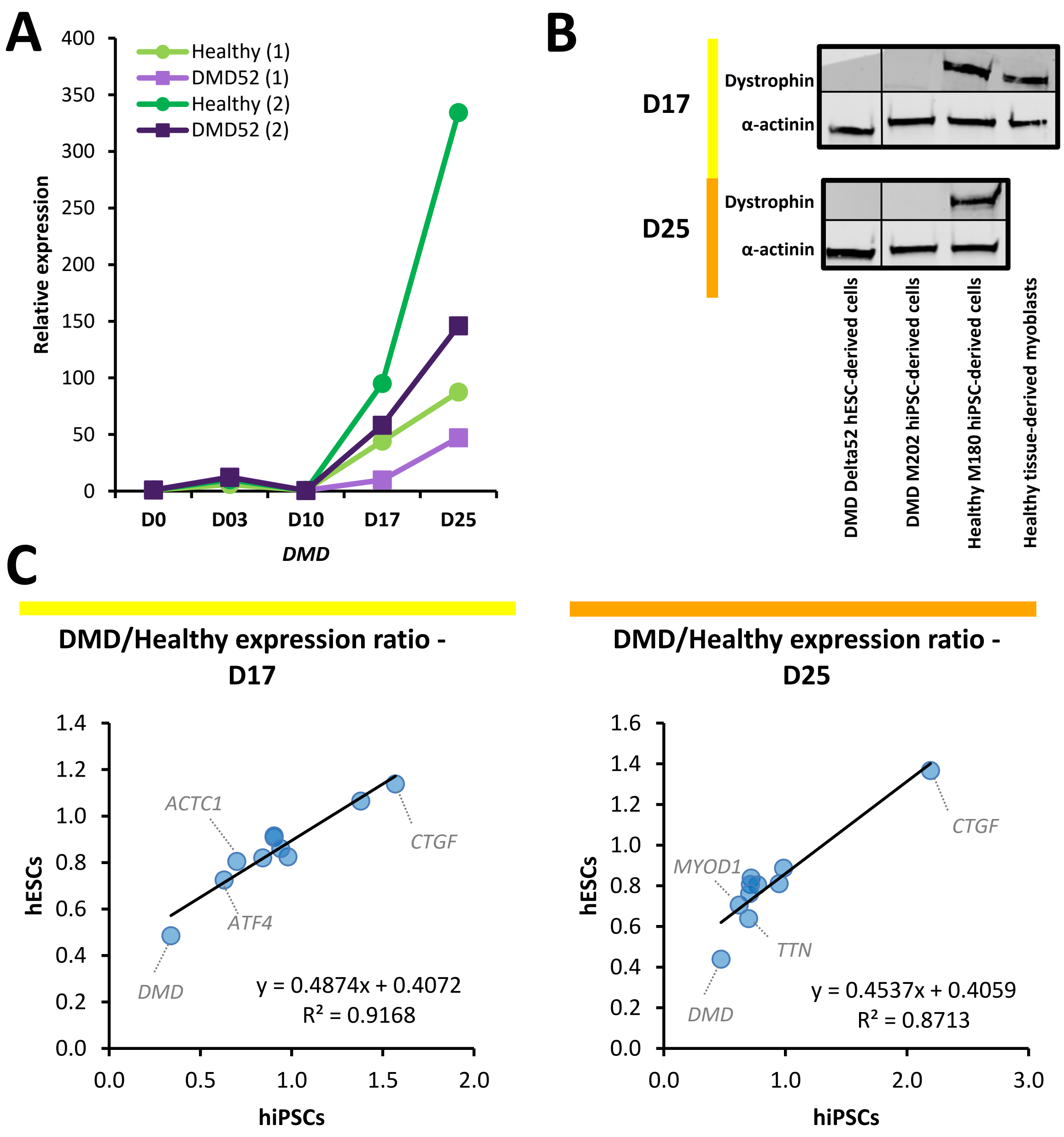

Figure S9 – Gene expression in the isogenic hESC-derived cells. A) qPCR quantification of *DMD* during 2 independent muscle differentiation (1 and 2) of both healthy and DMD Delta52 hESCs. **B)** Western blot of dystrophin at days 17 and 25. **C)** DMD/Healthy expression ratios in hiPSCs (RNA-seq quantification) versus hESCs (qPCR quantification) of 10 genes (*ACTC1*, *ATF4*, *APT5A1*, *CTGF*, *DMD*, *MYH3*, *MYOD1*, *MYOG*, *PCNA* and *TTN*) at day 17 and day 25.
