# Supplementary material for: Myogenesis modelled by human pluripotent stem cells: a multi‐omic study of Duchenne myopathy early onset
Source: J Cachexia Sarcopenia Muscle. 2021 Feb 14;12(1):209–32. doi: 10.1002/jcsm.12665 (PMC7890274; doi:10.1002/jcsm.12665)
Supplement: Supplementary file 17 — Figure S10. Supporting Information [file JCSM-12-209-s017.pdf]

Figure S10

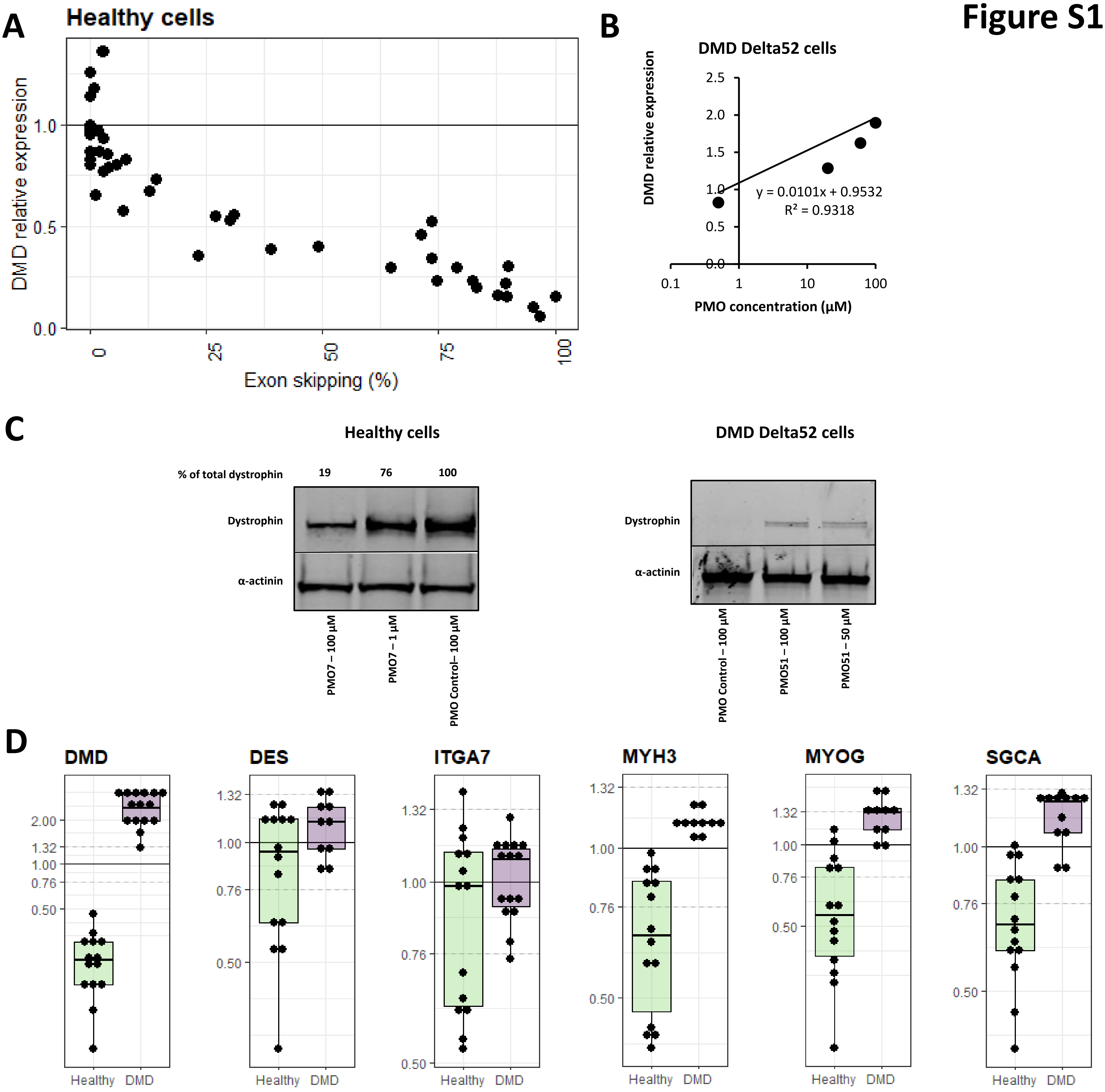

E

		Spearman correlation coefficient r		
DMD	Healthy	59 pairs	-0.88	****
	DMD	20 pairs	0.89	****
DES	Healthy	59 pairs	-0.06	
	DMD	12 pairs	0.31	
ITGA7	Healthy	59 pairs	-0.11	
	DMD	20 pairs	0.40	
MYH3	Healthy	59 pairs	-0.54	****
	DMD	12 pairs	0.89	***
MYOG	Healthy	59 pairs	-0.53	****
	DMD	12 pairs	0.67	*
SGCA	Healthy	56 pairs	-0.47	***
	DMD	14 pairs	0.75	**

Figure S10 – *DMD* knockdown and rescue at D17. qPCR quantification of *DMD* expression **A)** related to exon skipping efficiency (%) in healthy cells, and **B)** related to PMO concentration in DMD cells. **C)** Western blot quantification of dystrophin in M180 healthy cells (right) and DMD Delta52 cells (left). **D)** qPCR quantification of selected genes following exon skipping (boxplots of expression ratio of exon skipped/unskipped conditions when the exon skipping efficiency was above 70%). **E)** Spearman correlation coefficients per cell line between all skipped and unskipped conditions for each selected gene (*p-value ≤ 0.05, **p-value ≤ 0.01, ***p-value ≤ 0.001, ****p-value ≤ 0.0001).
