# Supplementary material for: Myogenesis modelled by human pluripotent stem cells: a multi‐omic study of Duchenne myopathy early onset
Source: J Cachexia Sarcopenia Muscle. 2021 Feb 14;12(1):209–32. doi: 10.1002/jcsm.12665 (PMC7890274; doi:10.1002/jcsm.12665)
Supplement: Supplementary file 18 — Figure S11. Supporting Information [file JCSM-12-209-s018.pdf]

Figure S11

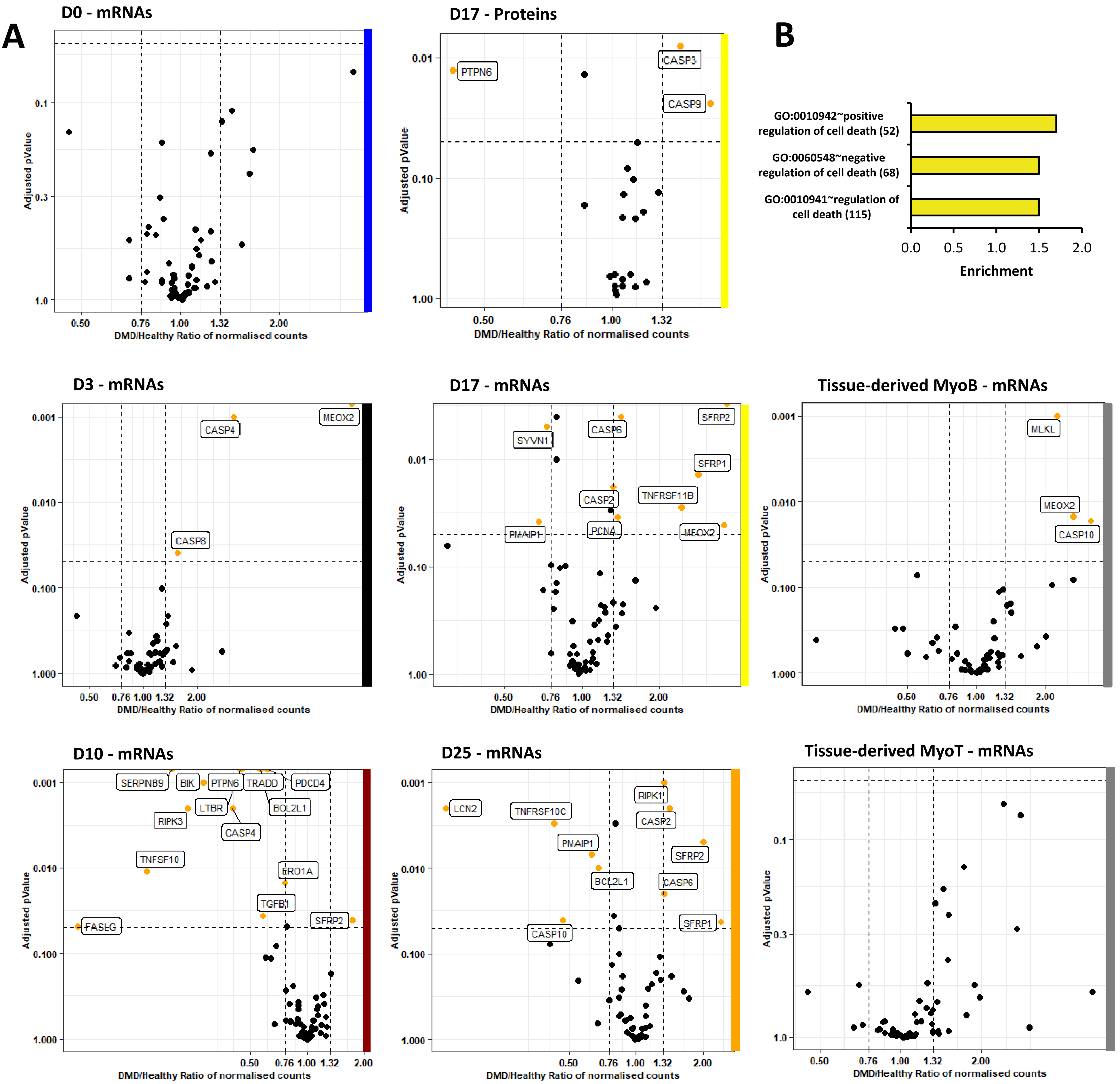

Figure S11 – Cell death marker genes in DMD versus healthy cells. A) Volcano plots of DMD/healthy comparisons during hiPSC- and tissue-derived cell differentiation. Statistical differences are symbolised with orange dots – vertical grey dashed lines represent DMD/Healthy ratio thresholds at 0.76 or 1.32 - the horizontal grey dashed line represents the adjusted p-value threshold at 0.05 (D: day). **B)** Gene ontology enrichment of significantly dysregulated terms in DMD hiPSC-derived myotubes. The number of genes involved in each term is indicated in brackets next to each GO term.
