# Supplementary material for: Myogenesis modelled by human pluripotent stem cells: a multi‐omic study of Duchenne myopathy early onset
Source: J Cachexia Sarcopenia Muscle. 2021 Feb 14;12(1):209–32. doi: 10.1002/jcsm.12665 (PMC7890274; doi:10.1002/jcsm.12665)
Supplement: Supplementary file 19 — Figure S12. Supporting Information [file JCSM-12-209-s019.pdf]

# Figure S12

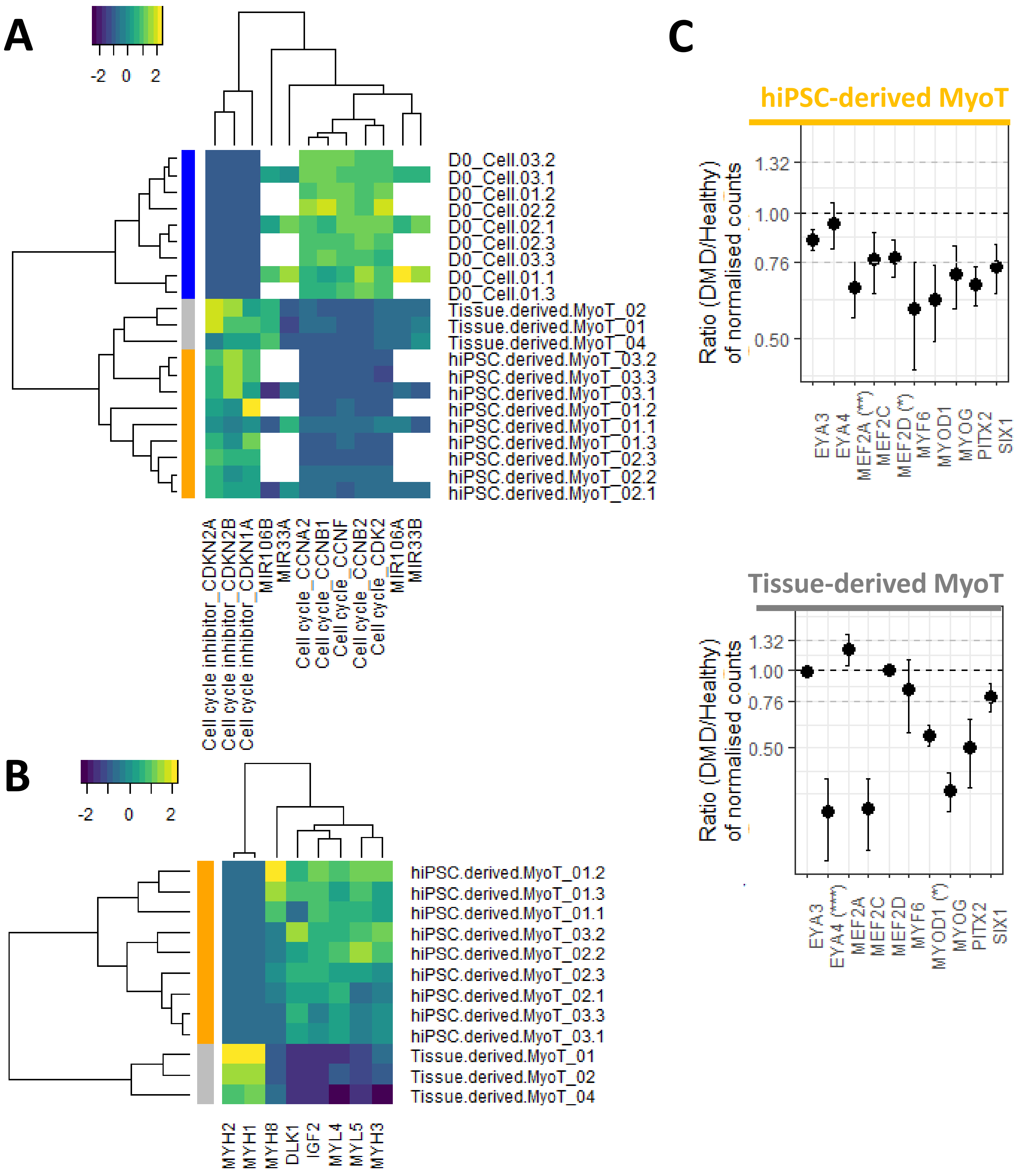


**Figure S12 – Comparison of hiPSC-derived and tissue-derived MyoT for the expression of cell cycle genes and myogenic regulators.** Hierarchical clustering and heatmap of **A)** selected cell cycle transcripts and miRNAs, and **B)** DLK1, IGF2 and selected myosin transcripts in hiPSCs (D0), hiPSC- and tissue-derived MyoT. **C)** Dotplot of DMD/healthy expression ratio of muscle transcription factors. Significant statistical differences are shown in brackets (*adjusted p-value ≤ 0.05, **adjusted p-value ≤ 0.01, ***adjusted p-value ≤ 0.001, ****adjusted p-value ≤ 0.0001). (hiPSC: human induced pluripotent stem cell; MyoT: myotube).
