# Supplementary material for: Myogenesis modelled by human pluripotent stem cells: a multi‐omic study of Duchenne myopathy early onset
Source: J Cachexia Sarcopenia Muscle. 2021 Feb 14;12(1):209–32. doi: 10.1002/jcsm.12665 (PMC7890274; doi:10.1002/jcsm.12665)
Supplement: Supplementary file 20 — Figure S13. Supporting Information [file JCSM-12-209-s020.pdf]

Figure S13

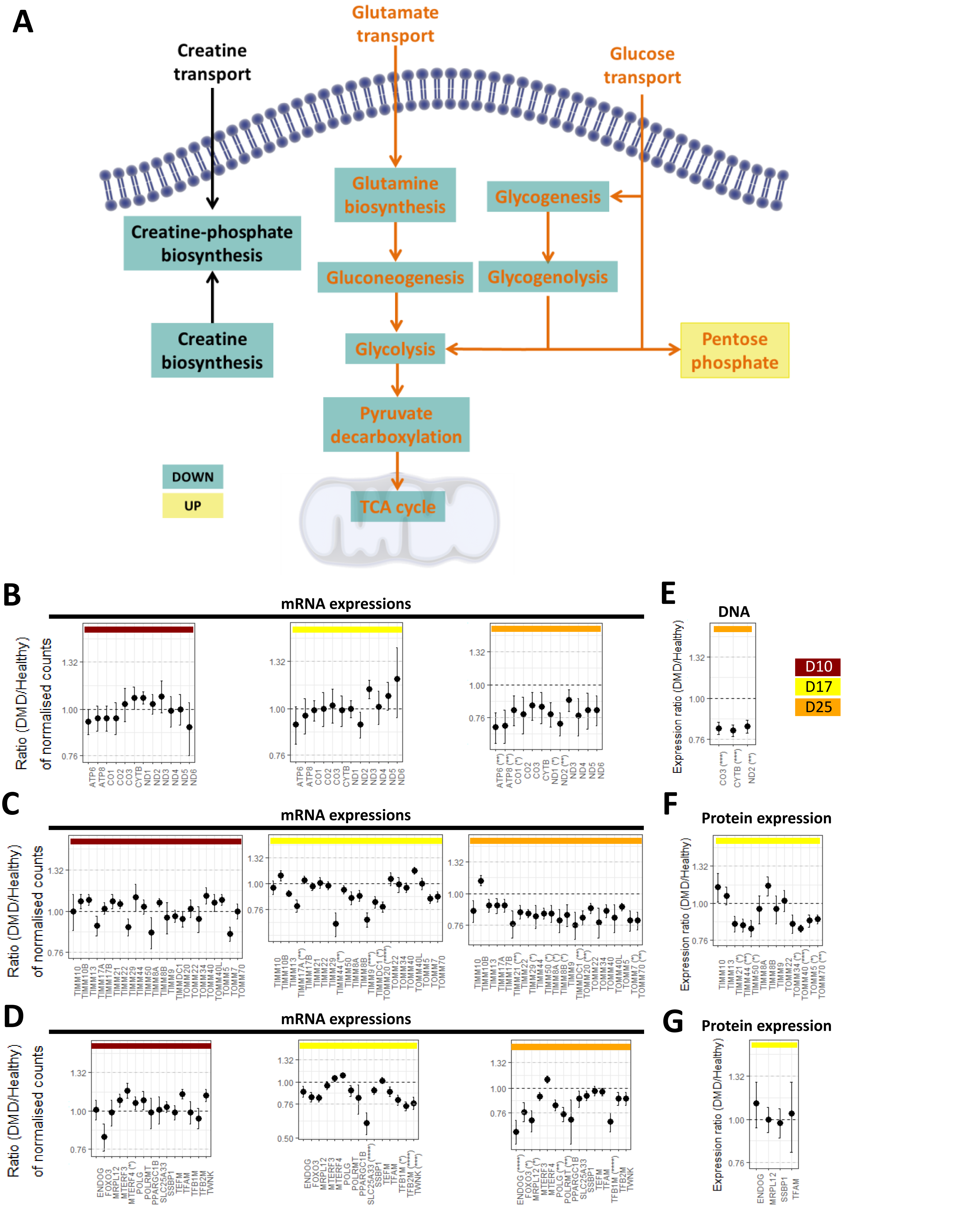

Figure S13 – Dysregulations of metabolic pathways and mitochondrial genes during differentiation of DMD hiPSCs. A) Scheme of metabolism dysregulations at day 25. Dotplots of **B)** mitochondrial transcripts, **C)** transcripts coding mitochondrial protein import, and **D)** transcripts coding mitochondrial transcription/replication; **E)** Mitochondrial DNA quantification by qPCR at D25. Dotplots of mitochondrial proteins expressed at D17 involved in **F)** protein import, **G)** mitochondrial transcription/replication. Statistics are in brackets (*adjusted p-value ≤ 0.05, **adjusted p-value ≤ 0.01, ***adjusted p-value ≤ 0.001, ****adjusted p-value ≤ 0.0001; D: day).
